# Effectiveness of monitoring mechanisms in reducing immunization dropouts among infants in health facilities of Maridi County, South Sudan

**DOI:** 10.1371/journal.pone.0356268

**Published:** 2026-09-03

**Authors:** Daniel Opinile Mark Igga, Nzomo Mwita, Kennedy Muni

**Affiliations:** 1 Amref International University, Juba, South Sudan; 2 Amref International University, Nairobi, Kenya; 3 World Health Organization South Sudan Country Office, Juba, South Sudan; IAVI, UNITED STATES OF AMERICA

## Abstract

**Introduction:**

Childhood immunization is among the most cost-effective public health strategies available, yet dropout rates, the proportion of infants who begin but fail to complete the recommended vaccination schedule, remain a persistent obstacle to achieving high and equitable coverage. Global third-dose pentavalent coverage stood at 86% in 2019, declined to 84% in 2020, and fell further to 82% in 2021, consistently falling short of the 85% minimum target. This shortfall is especially pronounced in fragile health systems such as that of South Sudan, where facility-level and community-level monitoring mechanisms vary widely in availability and quality.

**Methods:**

I conducted a prospective cohort study from 1 October to 31 December 2023 in Maridi County, Western Equatoria State, South Sudan. Fourteen health facilities were selected by stratified random sampling (seven from payams with active Boma Health Initiative [BHI] activities and seven from payams without). One vaccinator per facility was selected by coin toss, yielding 26 participants. A semi-structured interview guide administered in month one and a monthly observation checklist applied across all three months were used to collect data. Data were analyzed using descriptive statistics, chi-square tests, Spearman correlations, and multivariable regression to assess associations between monitoring mechanisms and facility-level dropout rates.

**Results:**

Three of the 14 facilities (21%) had no monitoring chart; 28.6% recorded a dropout rate above 10%, while half (50%) achieved the target of below 10%. Facilities with updated monitoring charts were significantly less likely to record high dropout rates (Spearman r = −0.725, p = 0.003). Boma Health Workers (BHWs), community-level health personnel who mobilize, trace, and refer immunization defaulters, were attached to 64% of facilities; 91.7% of facility-months in the low-dropout category had BHWs present, compared with 37.5% in the high-dropout category. Over the three months of observation, the proportion of facilities recording a dropout rate above 10% fell by 9.6 percentage points.

**Conclusions:**

Facility-based monitoring tools used by health workers (monitoring charts, immunization registers, and defaulter tracing systems) and community-based monitoring carried out by BHWs were each independently associated with lower dropout rates, and their combination appears to have a complementary effect. Neither mechanism was sufficient alone. We recommend that health workers use monitoring tools consistently and that the Boma Health Initiative be scaled up to health facilities currently without BHWs.

## Introduction

Immunization is one of the most efficient and successful health interventions for the reduction of child morbidity and mortality. The World Health Organization (WHO) established the Expanded Programme on Immunization (EPI) in 1974 to ensure universal access to routinely recommended childhood immunizations [1]. Despite half a century of progress under EPI, a substantial gap persists between the children who begin vaccination and those who complete it, a gap commonly measured as the immunization dropout rate.

In 2021, 18.2 million infants did not receive an initial dose of DTP vaccine globally, and an additional 6.8 million were only partially vaccinated. More than 60% of these under-immunized children live in ten countries spanning sub-Saharan Africa and South and South-East Asia, highlighting the geographical concentration of the challenge [2]. Monitoring data at subnational level is therefore critical to helping countries prioritize and tailor vaccination strategies to address immunization gaps.

Third-dose pentavalent coverage, a widely used proxy for immunization system performance, stood at 86% globally in 2019 but slipped to 84% in 2020 and 82% in 2021, consistently falling short of the 85% floor target set by the Immunization Agenda 2030 [3]. These setbacks, compounded by the disruption of routine health services during the COVID-19 pandemic, have renewed urgency around identifying which health system mechanisms can reliably reduce dropout.

South Sudan has been battling with underperformance and low coverage of the DPT-1 at 58% and DPT-3 at 50% in 2019. However, the country has experienced improvement in subsequent years and as of 2022, the coverage was at 97.6% for DPT-1 and 84.4% for DPT-3. Despite this progress, a dropout rate of 13.2% still signals that many children who start the immunization schedule do not complete it. Measles vaccine coverage was 42% in 2019 and 65.6% in 2022, still below the minimum performance target of 85% [4].

Maridi county has been among the top three counties leading in immunization performance in Western Equatoria State, despite the challenges of immunization defaulters that have been affecting the county performance from reaching its targets. According to 2022 programme data, third-dose pentavalent coverage in the county was 82% and measles vaccine coverage was 55%, both below the national 85% target. Prompt action and a collaborative approach linking facility-level monitoring with community-level tracing are needed to ensure infants complete their vaccination schedules as per national guidelines, thereby increasing herd immunity and reducing the burden of vaccine-preventable disease.

The Immunization Coverage Monitoring Chart is a very simple yet effective tool that has been in use for decades. When updated monthly, it gives a visual representation that enables immunization programme managers to set and evaluate monthly goals in a timely fashion, provides a basis for comparing performance across different time periods, monitors each vaccine and dose separately, and reflects the situation accurately [5]. Alongside the monitoring chart, the defaulter tracing system, which is centred on the tickler file (a card-based system organized by month of next expected visit), gives health workers an operational means of identifying which specific children have missed an appointment. Community-level follow-up in Maridi County is supported by the Boma Health Initiative (BHI), a South Sudan-specific programme that deploys trained Boma Health Workers (BHWs) at village level to mobilize communities, counsel mothers, and refer immunization defaulters back to health facilities.

Because the term is used inconsistently in the immunization literature, we define it explicitly here. For the purposes of this study, *monitoring mechanisms* refers to the systematic tools and processes used to track vaccination coverage, identify defaulters, and prompt corrective action. These fall into two categories, distinguished by who operates them. *Facility-level monitoring* is carried out by vaccinators and health workers inside the health facility, using the immunization monitoring chart, immunization registers, defaulter tracing cards and tickler files, and the health facility microplanning (HFMP) document. *Community-level monitoring* is carried out by BHWs in the facility catchment area, and consists of household mobilization, tracing of children identified as defaulters, and referral of those children back to the facility. This distinction is maintained throughout the paper.

Little prospective evidence exists from South Sudan, or from comparable fragile-state settings, on whether these mechanisms actually reduce dropout rates when implemented in real-world health facilities. This study sought to fill that gap by following 14 health facilities across three months, comparing facilities with and without BHI activities, and examining the associations between monitoring chart use, BHW engagement, defaulter tracing system functionality, and measured dropout rates.

## Materials and methods

### Study design and setting

A prospective cohort study design was used, for a duration of three months from 01/10/2023 to 31/12/2023, in order to answer the research questions. The unit of analysis was the health facility. The study was carried out in Maridi County of Western Equatoria State, South Sudan, approximately 86 miles from Yambio (the state capital) and 185 miles from Juba. The county has five payams and 81 bomas. Health facilities were divided into two strata: one with boma health initiative activities and those that do not have these activities in order to determine the effectiveness of immunization monitoring mechanisms to reduce immunization dropouts.

### Sampling

A stratified random sampling method was used to draw participants and the health facilities from the two strata. Using EPI info software for calculating the samples needed for the study, at 95% confidence level, 14 health facilities were selected. In the 14 health facilities, a total of 26 vaccinators were divided proportionately into 2 categories: 13 vaccinators from health facilities with BHI and 13 vaccinators from health facilities with no BHI. A coin was tossed to select a vaccinator from each eligible health facility (each facility generally has two vaccinators).

Inclusion criteria: Health facilities were selected based on their functionality and provision of immunization services at the health facility. Each health facility had two vaccinators who relatively performed same task, thus one vaccinator per health facility was included and assessed using the semi-structured interview, selected by the coin toss described above.

Exclusion criteria: Health facilities that did not provide immunization service were excluded from sampling. The vaccinator not selected by the coin toss was excluded from the semi-structured interview. The study excluded participants who were minors, aged < 18 years.

### Data collection

A semi-structured interview guide and an observation checklist were used to collect data from the respondents. The semi-structured interview guide had a written informed consent that explained the purpose of the research and how their responses would be confidentially kept by the research team. Participants were free to leave the research at any given point in time within the research period. Each participant had to sign the consent form to indicate their acceptance and willingness to participate in the study.

The consent form was prepared in English. Because participants varied in the language they were most comfortable with, I verbally interpreted the consent form into each participant’s preferred language (Juba Arabic, Muru, Azande, Baka, or Avokaya) immediately before they signed. No written translations were produced; the interpretation was delivered by me, in person, on each occasion, and I obtained written informed consent from every participant myself. This verbal interpretation protocol was described in the study protocol and approved by the Amref International University Ethics and Scientific Review Committee and the South Sudan Ministry of Health Research Ethics Review Board.

Whereas the observation checklist was used to collect data from the health facility using the approval letter from the county health department for data collection at the health facility. No participant was involved in the data collected through the observation checklist. The checklist items were transcribed from records the facility maintains for its own reporting (the monthly summary report, the immunization registers, and the monitoring chart) rather than from real-time observation of health worker behaviour. Because those records document performance accumulated across the whole month, rather than conduct at the moment of the visit, this reduces (though it does not eliminate) the risk that facilities appeared to perform better simply because they knew I was present. No independent re-abstraction of the records was carried out, and this is noted as a limitation.

### Data management and analysis

Data was collected on the first month using the semi-structured interview guide while observation checklist was used in the three months, from 01/10/2023 to 31/12/2023, hence data was collected on monthly basis for the duration of the study. The data from the research instrument was cleaned, coded, and entered into Excel sheet on monthly basis for analysis as well as tracking the study and into SPSS after the study duration for further analysis. Descriptive statistics such as frequencies and percentages were used to describe the respondents’ demographic statistics, marital status, educational level and years of experience in expanded programme on immunization. Whereas bivariable analysis was used to determine relationships between the independent variables and the dependent variable.

Chi square test was used to compare the dropout rates of health facilities that were exposed to and those not exposed to boma health initiative, immunization monitoring chart and linking defaulters for vaccination. This examined whether the presence of BHWs in health facilities was associated with low dropout rates (less than 10%) or high dropout rates (more than 10%). Multivariable regression analysis was used to determine association between the variables to answer the research questions and hypotheses. Because facilities were not randomized to condition, all results reported below are associations and should not be read as establishing causation. Statistical significance was set at α = 0.05.

### Ethical considerations

The study obtained ethical approval from the AMIU ESRC, South Sudan Research Ethics Committee, approval from the state ministry of health in Western Equatoria State and from Maridi County Health Department that oversees the health facilities where the research was conducted.

I obtained written informed consent from every participant before data collection began, having first explained the purpose of the research and assured them of the confidentiality measures that would be undertaken to safeguard respondent data. All the research data were kept safely by me for future reference, identified by participant number rather than name.

## Results

### Participant and facility characteristics

All 14 health facilities that were selected for the study were reached during the study. Fourteen interviews were completed in month one and 42 observation checklists were collected (one per facility per month). Out of the 14 health facilities, 12 (86%) were supported by non-governmental organizations while 2 (14%) were supported by the Government. Throughout this section, “respondent” denotes the single vaccinator selected to respond on behalf of each health facility; there were therefore 14 respondents, one per facility. Eleven respondents (79%) were male and 3 (21%) were female; 13 (93%) were married. Ten respondents (71%) had reached secondary level of education and 4 (29%) primary level. Disaggregated by sex, 10 male respondents had attained secondary-level education and 1 attained primary education level, while all 3 female respondents had attained primary-level education only. Educational level, with confidence interval (Mean difference: 1.71, 95% CI:1.44–1.98) is a significant factor for using immunization tools (Table 1).

**Table 1:** Demographic characteristics of respondents (N = 14).

Characteristic	n	%
**Sex**		
Male	11	78.6
Female	3	21.4
**Marital status**		
Married	13	92.9
Single	1	7.1
**Highest education level**		
Secondary	10	71.4
Primary	4	28.6
**Health facility funding source**		
NGO-supported	12	85.7
Government-supported	2	14.3

NGO = non-governmental organization

### Use of immunization monitoring charts to track immunization performance

Eleven out of fourteen facilities (79%) health facilities had monitoring chart for displaying the immunization performance of the health facility, with 10 of those 11 (71%) of the health facilities having their monitoring charts updated with the previous month data. The remaining 3 facilities (21%) had no monitoring chart at all. Table 2 cross-tabulates monitoring chart status with dropout rate category across the 42 facility-month observations.

**Table 2:** Monitoring chart status by dropout rate category across 42 facility-month observations.

Monitoring chart status	Dropout <10% n (%)	Dropout >10% n (%)	No chart n (%)
Chart present and updated	24 (57.1)	4 (9.5)	n/a
Chart present, not updated	0	5 (11.9)	n/a
No monitoring chart	n/a	n/a	9 (21.4)

Values are counts and column percentages. Cells marked n/a indicate structural absence. Rows are mutually exclusive.

Respondents were not restricted to a single answer, and several facilities used more than one tool; the categories below therefore overlap and do not sum to 100%. Seven respondents (50%) reported using immunization registers to identify immunization defaulters, 4 (29%) used tickler files, 2 (14%) used defaulter tracing cards, and 1 (7%) used the monitoring chart to identify defaulters. Use of monitoring tools to identify immunization defaulters was significantly associated with a lower rate of defaulters (Mean difference: 1.78, 95% CI: 1.22–2.35).

The use of monitoring tools was associated with the health facility microplanning document having been updated, with a 54% coefficient of association and p-value 0.04, indicating that the more the microplanning documents were updated the more likely monitoring tools were used to identity immunization defaulters. The observation data indicated that most of the health facility filled the immunization registers correctly regardless of the dropout rate. This is a very important aspect of the data management as incorrect data filling would result into misclassification of the dropouts and also immunization visits.

Eleven out of the fourteen of the respondents (79%) confirmed that they used monitoring chart for plotting the performance of the 1st and 3rd dose of Pentavalent, 13 (93%) recorded child’s information in the immunization register, child health card and dropout/defaulter tracing cards at the start of the vaccination session. Eight respondents (57%) check their monitoring chart three (3) times in a quarter while 2 (14%) checked twice and 3 (21%) checked once. Updated monitoring chart showed significant inverse association with the dropout rate of the health facility with 72.5% correlation coefficient and p-value of 0.003, implying that the more a health facility updated their monitoring chart the less likely would the health facility have increased dropout rate.

### Involvement of Boma Health Initiative contributes to reduced immunization dropouts

Sixty-four percent (9 of 14) of the respondents affirmed that they have boma health workers attached to their facilities while 5 (36%) had community mobilizers who were volunteers assigned by their community. Eighty six percent (12 of 14) of the health workers and vaccinators affirmed that they received referrals from the BHWs and community mobilizers while 2 (14%) of the respondents did not receive referrals, indicating gaps in referral activities. The type of referrals is important to address the dropouts: out of the two types of referrals, defaulters and zero-dose referrals, heath facilities with less than 10% dropout rate had 86% of the received referrals were defaulters, whereas health facilities with more than 10% dropout rate had 50% defaulters, 25% zero-dose and 25% no referrals.

Table 3 presents the relationship between BHW attachment and dropout rate category.

**Table 3:** BHW attachment status in relation to dropout rate category across 42 facility-month observations.

BHW status	Dropout <10% n (%)	Dropout >10% n (%)	No monitoring chart n (%)
BHW attached	22 (91.7)	3 (37.5)	6 (60.0)
No BHW	2 (8.3)	5 (62.5)	4 (40.0)
**Total**	**24**	**8**	**10**

BHW = Boma Health Worker. Values are counts and row percentages.

Facilities with BHWs attached accounted for 91.7% of low-dropout facility-months, compared with 37.5% of high-dropout facility-months. The number of BHWs attached to a facility was significantly associated with lower dropout (Mean difference: 2.35, 95% CI: 2.07–2.64).

All the health facilities with desirable (less than 10%) dropout rate had put effort to identify the defaulters and trace them in order to complete their vaccination schedule while facilities with more than 10% dropout rates had documented effort to identify and trace defaulters recorded in only 50% of their facility-month observations; that is, in half of the months observed at those facilities, no such effort was recorded at all. The checklist captured this as a binary yes/no item and did not measure how much effort was expended.

### Defaulter tracing system to ensure infants complete their vaccination schedule

Ninety three percent (13 of 14) of the respondents confirmed that their facilities have defaulter tracing system, with eighty-six percent (12 of 14) who filled the dropout cards, thirty-six percent (5 of 14) knew the overall importance of using tickler file as a defaulter tracing system while twenty-one percent (3 of 14) only knew that it is used to track the next visit of the infant. Thirteen respondents (93%) understood that vaccine stockouts increase the number of defaulters and dropouts. These findings are summarized in Table 4.

**Table 4:** Defaulter tracing system characteristics and related practices (N = 14 facilities).

Variable	n	%
Health facility has a defaulter tracing system	13	92.9
Defaulter cards filled during vaccination sessions	12	85.7
Respondent knows full purpose of tickler file	5	35.7
Vaccine stockout recognized as a cause of dropout	13	92.9
Health facility microplanning document updated	13	92.9

HFMP = Health Facility Microplanning document.

A health facility without defaulter tracing system is more likely to have increased dropout rate than one with defaulter tracing system (Mean difference: 1.28, 95% CI: 1.02–1.56). About fifteen percent (2 out of 13, 15%) of health facilities who had health facility microplanning document did not agree that defaulter tracing system is important to reduce the number of infants who did not come on time for vaccination.

Across the three months of observation, the proportion of facilities recording a dropout rate above 10% fell by 9.6 percentage points.

## Discussion

### Monitoring charts and the value of routine data use

Facilities that had a monitoring chart, and particularly those that kept it updated, were less likely to record a dropout rate above 10% (r = −0.725, p = 0.003). Because facilities were not randomized, this is an association rather than a demonstrated cause. Our data are consistent with the findings of Chantler and colleagues [6], who showed that the high dropout rates in the health facility were associated with immunization activities and routine immunization registers that were not updated routinely, and defaulter tracing that was rarely conducted. The findings are also consistent with those of Shikuku et al. [7], who showed that active analysis and utilizing routine immunization data together with investing in community health strengthening systems improve immunization coverage.

Prakash et al. [8] concluded that comprehensive record-keeping and robust support systems for community health workers are pivotal to improving immunization coverage and reducing the burden of vaccine-preventable diseases; our data are consistent with that conclusion in the South Sudanese context. Health workers should be able to properly use the monitoring tools such as registers and monitoring charts which are vital in ensuring improvement is achieved in immunization thus reducing dropouts in the community. It is worth noting here that the monitoring chart is a performance tool: it is designed to display aggregate coverage and gauge the success of the immunization programme, rather than to identify individual defaulters, for which the register and the defaulter tracing card are the appropriate instruments. Most respondents used the tools in exactly this way, which is a positive indicator of monitoring tool literacy and one worth reinforcing in supervision and training.

### Boma Health Workers and the community interface

Facilities where health workers made documented efforts to identify defaulters were associated with lower dropout rates (Spearman r = 0.354, p = 0.021). This measure requires an important caveat: the observation checklist recorded only whether any effort to identify and link defaulters occurred in a given month, as a binary yes/no item. It did not quantify how much effort was expended, how many households were visited, or how many contacts were attempted, and we did not collect data that would support such a measure. We therefore make no claim about the amount of effort involved, and the earlier framing of this result as a 66% reduction in dropout has been withdrawn as an overinterpretation. Our data are consistent with the findings of Samuel [9], who showed that effective referrals from the community for missed immunization services were due to strengthened linkages between communities and health centers. The mechanism we observed is a two-sided one: health workers identify defaulters and hand over a list, and BHWs locate those children in the community and refer them back. Health workers reported feeling supported in tracing defaulters when BHWs were available to help find the infants. This suggests that strengthening either side of the community-facility interface in isolation may yield diminishing returns.

The type of referral also distinguished the two groups. Low-dropout facilities received predominantly defaulter referrals (86%), whereas high-dropout facilities received a more fragmented mix of defaulter, zero-dose, and absent referrals. This suggests that BHW follow-up is most effective for dropout reduction when it is directed at children already known to have missed a dose, while still ensuring zero-dose children are captured.

### Microplanning, defaulter tracing, and vaccine supply

A positive association between the last time Health Facility Microplanning document was updated, the health worker’s ability to use the document and with performance monitoring chart was observed. These indicate the more frequent the HFMP document could be updated, the more likely the health workers and vaccinator would be able to use the document. The study is consistent with Ali et al. [10], who showed that effective routine immunization depends on a microplanning that requires accurate estimation of the population and location maps that shows the location of health facilities and villages of the target populations.

Although Mafigiri et al [11] findings showed that lack of updated microplan in the health facility was attributed to lack of knowledge, some health facilities with no updated microplans had high immunization coverage and concluded that health workers’ misunderstanding and limited knowledge about the microplanning process, especially at peripheral health facilities, coupled with the complex, bulky nature of the microplanning tool, reduces the effectiveness of microplanning in improving routine immunization. On the contrary, updating HFMP document was supported by the county health department and updated copies of the HFMP document were handed to the health facility for implementation, thus eliminating the risk that health workers would update the document by themselves.

Vaccine supply deserves specific comment, since 93% of respondents identified stockouts as a driver of dropout. Vaccine quantities for a facility are forecast from the catchment population estimate held in the HFMP document and from monthly consumption recorded in the registers, which are the same records that feed the monitoring chart. Where those records are not maintained, ordering is weakened at the same time as defaulter identification is, which may partly explain why the two travelled together in our data. A child turned away during a stockout becomes a defaulter like any other, and should in principle be entered on a defaulter tracing card and recalled once supply is restored. We did not measure whether such recall actually occurred, and this is a gap that future work should address.

### Limitations

Several limitations should be considered. The sample of 14 facilities limits statistical power and generalizability beyond Maridi County. Interviews were conducted at a single time point, introducing potential social desirability bias. The observation checklist was completed by me during facility visits; although it drew on monthly records rather than real-time behaviour, the possibility that facilities performed better in observed months cannot be excluded, and no independent re-abstraction was carried out. Finally, because facilities were not randomized to condition, the associations reported here cannot establish causation.

## Conclusions

Facility-level monitoring (the monitoring chart, the immunization register, and the defaulter tracing system, operated by vaccinators and health workers) and community-level monitoring, operated by BHWs, were each associated with lower immunization dropout rates over the three-month study period. Neither was sufficient alone. Three facilities had no monitoring chart at all, and facilities without BHWs accounted for the majority of high-dropout observations.

The microplanning document though not directly a monitoring tool, supported to ensure that vaccine quantities for the health facility were sufficient. Lack of vaccine supply in the health facility was identified to be one of the causes infants’ dropouts of their vaccination schedule. The study observed that lack of efforts from health workers to identify and link defaulters, lack of updated monitoring charts and incorrectly filled immunization registers, absence of BHWs in the health facilities, no referrals from BHWs and lack of support from concerned organizations and government were associated with high dropout rates in some of the health facilities of Maridi County.

Therefore, Policy makers to leverage on the defaulter tracing system in the health facilities and activate them to support the effort of the health workers in identifying infants who defaulted easily and improve tracking of the defaulters, the government and its partners to prioritize scaling the BHI to health facilities without BHWs is paramount to support narrowing the gap of defaulters in the community thus reducing immunization defaulters in the community. We further recommend that monitoring data, including HFMP population estimates, register consumption figures, and monthly coverage summaries, be linked directly to vaccine ordering, so that stockout-driven dropout becomes a predictable and preventable event rather than a recurring disruption.

## Supporting information

S1 Table Organizational support to the health facility. Frequency and percentage distribution of the type of organization supporting the surveyed health facilities (N = 14): non-governmental organization (NGO) versus government.
(DOCX)

S2 Table Gender of respondents. Frequency and percentage distribution of respondent gender (N = 14): male versus female.
(DOCX)

S3 Table Marital status of respondents. Frequency and percentage distribution of respondent marital status (N = 14): single versus married.
(DOCX)

S4 Table Educational level of respondents. Frequency and percentage distribution of respondent educational attainment (N = 14): primary versus secondary.
(DOCX)

S5 Table Health facilities with a monitoring chart. Frequency and percentage distribution of health facilities reporting whether they maintain an immunization monitoring chart (N = 14).
(DOCX)

S6 Table Monitoring tool used to identify immunization defaulters. Frequency and percentage distribution of the tool used by health facilities to identify immunization defaulters (N = 14): immunization register, tickler file, defaulter tracing cards, or monitoring chart.
(DOCX)

S7 Table Accuracy of immunization register completion, by facility dropout-rate category. Cross-tabulation of whether immunization registers were correctly filled for the preceding month against facility dropout-rate category (no monitoring chart, < 10%, > 10%; N = 42).
(DOCX)

S8 Table Monitoring chart updated with the previous month’s data. Frequency and percentage distribution of whether the monitoring chart was updated with the previous month’s data (N = 14).
(DOCX)

S9 Table Frequency of quarterly performance review using the monitoring chart. Frequency and percentage distribution of how many times per quarter health facility staff check performance using the monitoring chart (N = 14).
(DOCX)

S10 Table Correlation between monitoring chart updates and facility dropout rate. Spearman’s rank correlation between facility dropout-rate classification and whether the monitoring chart was updated with the previous month’s data (N = 14; rho = −0.725, p = .003).
(DOCX)

S11 Table Accuracy of monitoring chart plotting, by facility dropout-rate category. Cross-tabulation of whether the monitoring chart was plotted correctly against facility dropout-rate category (no monitoring chart, < 10%, > 10%; N = 42).
(DOCX)

S12 Table Health facilities with boma health workers attached. Frequency and percentage distribution of health facilities reporting whether boma health workers (BHWs) are attached to the facility (N = 14).
(DOCX)

S13 Table Receipt of immunization referrals from boma health workers. Frequency and percentage distribution of whether health facility staff received immunization referrals from boma health workers or community mobilizers (N = 14).
(DOCX)

S14 Table Type of immunization referral received from boma health workers, by facility dropout-rate category. Cross-tabulation of the type of referral received (defaulters’ referral, zero-dose referral, both, or none) against facility dropout-rate category (no monitoring chart, < 10%, > 10%; N = 14).
(DOCX)

S15 Table Presence of boma health workers in relation to facility dropout rate. Cross-tabulation of whether boma health workers (BHWs) are present at the facility against facility dropout-rate category (no monitoring chart, < 10%, > 10%; N = 42).
(DOCX)

S16 Table Health care worker follow-up of identified defaulters, by facility dropout-rate category. Cross-tabulation of whether health care workers (HCWs) made an effort to link with and trace identified defaulters against facility dropout-rate category (no monitoring chart, < 10%, > 10%; N = 42).
(DOCX)

S17 Table Correlation between health care worker defaulter tracing and facility dropout rate. Spearman’s rank correlation between whether HCWs made an effort to trace identified defaulters and facility dropout rate (N = 42; rho = 0.354, p = .021).
(DOCX)

S18 Table Counseling of defaulting mothers by boma health workers. Frequency and percentage distribution of whether boma health workers counsel mothers of defaulting infants to complete the vaccination schedule before age 1 (N = 14).
(DOCX)

S19 Table Health facilities with a defaulter tracing system. Frequency and percentage distribution of whether the health facility has a defaulter tracing system in place (N = 14).
(DOCX)

S20 Table Frequency of reviewing the tickler file for defaulter information. Frequency and percentage distribution of how often staff collect defaulter information from the tickler file (N = 14): none, daily, weekly, or monthly.
(DOCX)

S21 Table Perceived consequence of absent defaulter tracing systems on vaccine completion. Frequency and percentage distribution of respondent agreement with the statement that, absent a defaulter tracing system, children are less likely to complete vaccine doses such as penta-3 or measles (N = 14).
(DOCX)

S22 Table Health facilities with documented immunization microplanning. Frequency and percentage distribution of whether the health facility has documented microplanning specifying outreach locations, stakeholders, a sketch map, and an analysis of the local immunization situation (N = 14).
(DOCX)

S23 Table Recency of health facility microplanning updates. Frequency and percentage distribution of when the health facility’s immunization microplanning was last updated: less than one year ago, one year ago, or 2–3 years ago (N = 14).
(DOCX)

S24 Table One-way ANOVA of facility indicators across dropout-rate groups. One-way analysis of variance testing whether four facility-level indicators differ across dropout-rate group (no monitoring chart, < 10%, > 10%; N = 14): whether the monitoring chart is updated with the previous month’s data, F(2,11) = 12.833, p = .001; whether the health facility has a defaulter tracing system, F(2,11) = 0.458, p = .644; whether immunization referrals were received from boma health workers or community mobilizers, F(2,11) = 3.929, p = .052; and the recency of the last microplanning update, F(2,11) = 4.939, p = .029.
(DOCX)

S1 Fig One-sample t-tests of observation-based indicators. SPSS one-sample t-test output (test value = 0) for five facility-level indicators: whether the monitoring chart was updated with data from the monthly summary report, whether the chart was plotted correctly, whether immunization registers were filled correctly for the preceding month, whether health care workers made an effort to link and trace identified defaulters, and dropout rate. Reports t, df, two-tailed significance, mean difference, and the 95% confidence interval of the difference (df = 41 for each test).
(PNG)

S2 Fig Monitoring chart updates in relation to facility dropout rate. SPSS cross-tabulation and chi-square output for whether the monitoring chart was updated with data from the monthly summary report against facility dropout-rate category (no monitoring chart, < 10%, > 10%; N = 42; Pearson χ² = 42.000, df = 2, p < .001).
(PNG)

S3 Fig Health care worker defaulter tracing in relation to facility dropout rate. SPSS cross-tabulation and chi-square output for whether health care workers made an effort to link and trace identified defaulters against facility dropout-rate category (no monitoring chart, < 10%, > 10%; N = 42; Pearson χ² = 14.348, df = 2, p < .001).
(PNG)

S4 Fig Receipt of boma health worker referrals in relation to facility dropout rate. SPSS cross-tabulation and chi-square output for whether health facility staff received immunization referrals from boma health workers or community mobilizers against facility dropout-rate category (no monitoring chart, < 10%, > 10%; N = 14; Pearson χ² = 5.833, df = 2, p = .054).
(PNG)
