# Supplementary material for: Effectiveness of monitoring mechanisms in reducing immunization dropouts among infants in health facilities of Maridi County, South Sudan
Source: PLoS One. 2026 Sep 3;21(9):e0356268. doi: 10.1371/journal.pone.0356268 (PMC13541179; doi:10.1371/journal.pone.0356268)
Supplement: Supplementary file 1 [file pone.0356268.s001.docx]

**S1 Table. Organizational support to the health facility.**

Frequency and percentage distribution of the type of organization supporting the surveyed health facilities (N = 14): non-governmental organization (NGO) versus government.

**Who supports the health facility**

| **Response** | **Frequency** | **Percent** | **Valid Percent** | **Cumulative Percent** |
| --- | --- | --- | --- | --- |
| NGO | 12 | 85.7 | 85.7 | 85.7 |
| Government | 2 | 14.3 | 14.3 | 100.0 |
| **Total** | **14** | **100.0** | **100.0** |  |
