# Supplementary material for: Effectiveness of monitoring mechanisms in reducing immunization dropouts among infants in health facilities of Maridi County, South Sudan
Source: PLoS One. 2026 Sep 3;21(9):e0356268. doi: 10.1371/journal.pone.0356268 (PMC13541179; doi:10.1371/journal.pone.0356268)
Supplement: Supplementary file 2 [file pone.0356268.s002.docx]

**S2 Table. Gender of respondents.**

Frequency and percentage distribution of respondent gender (N = 14): male versus female.

**Gender of the respondent**

| **Response** | **Frequency** | **Percent** | **Valid Percent** | **Cumulative Percent** |
| --- | --- | --- | --- | --- |
| Male | 11 | 78.6 | 78.6 | 78.6 |
| Female | 3 | 21.4 | 21.4 | 100.0 |
| **Total** | **14** | **100.0** | **100.0** |  |
