# Supplementary material for: Effectiveness of monitoring mechanisms in reducing immunization dropouts among infants in health facilities of Maridi County, South Sudan
Source: PLoS One. 2026 Sep 3;21(9):e0356268. doi: 10.1371/journal.pone.0356268 (PMC13541179; doi:10.1371/journal.pone.0356268)
Supplement: Supplementary file 3 [file pone.0356268.s003.docx]

**S3 Table. Marital status of respondents.**

Frequency and percentage distribution of respondent marital status (N = 14): single versus married.

**Marital status of the respondent**

| **Response** | **Frequency** | **Percent** | **Valid Percent** | **Cumulative Percent** |
| --- | --- | --- | --- | --- |
| Single | 1 | 7.1 | 7.1 | 7.1 |
| Married | 13 | 92.9 | 92.9 | 100.0 |
| **Total** | **14** | **100.0** | **100.0** |  |
