# Supplementary material for: Effectiveness of monitoring mechanisms in reducing immunization dropouts among infants in health facilities of Maridi County, South Sudan
Source: PLoS One. 2026 Sep 3;21(9):e0356268. doi: 10.1371/journal.pone.0356268 (PMC13541179; doi:10.1371/journal.pone.0356268)
Supplement: Supplementary file 4 [file pone.0356268.s004.docx]

**S4 Table. Educational level of respondents.**

Frequency and percentage distribution of respondent educational attainment (N = 14): primary versus secondary.

**Educational level of the respondent**

| **Response** | **Frequency** | **Percent** | **Valid Percent** | **Cumulative Percent** |
| --- | --- | --- | --- | --- |
| Primary | 4 | 28.6 | 28.6 | 28.6 |
| Secondary | 10 | 71.4 | 71.4 | 100.0 |
| **Total** | **14** | **100.0** | **100.0** |  |
