# Supplementary material for: Effectiveness of monitoring mechanisms in reducing immunization dropouts among infants in health facilities of Maridi County, South Sudan
Source: PLoS One. 2026 Sep 3;21(9):e0356268. doi: 10.1371/journal.pone.0356268 (PMC13541179; doi:10.1371/journal.pone.0356268)
Supplement: Supplementary file 6 [file pone.0356268.s006.docx]

**S6 Table. Monitoring tool used to identify immunization defaulters.**

Frequency and percentage distribution of the tool used by health facilities to identify immunization defaulters (N = 14): immunization register, tickler file, defaulter tracing cards, or monitoring chart.

**Monitoring tool used to identify immunization defaulters**

| **Response** | **Frequency** | **Percent** | **Valid Percent** | **Cumulative Percent** |
| --- | --- | --- | --- | --- |
| Immunization register | 7 | 50.0 | 50.0 | 50.0 |
| Tickler file | 4 | 28.6 | 28.6 | 78.6 |
| Defaulter tracing cards | 2 | 14.3 | 14.3 | 92.9 |
| Monitoring chart | 1 | 7.1 | 7.1 | 100.0 |
| **Total** | **14** | **100.0** | **100.0** |  |
