# Supplementary material for: Effectiveness of monitoring mechanisms in reducing immunization dropouts among infants in health facilities of Maridi County, South Sudan
Source: PLoS One. 2026 Sep 3;21(9):e0356268. doi: 10.1371/journal.pone.0356268 (PMC13541179; doi:10.1371/journal.pone.0356268)
Supplement: Supplementary file 8 [file pone.0356268.s008.docx]

**S8 Table. Monitoring chart updated with the previous month's data.**

Frequency and percentage distribution of whether the monitoring chart was updated with the previous month's data (N = 14).

**Is the monitoring chart updated with previous month data?**

| **Response** | **Frequency** | **Percent** | **Valid Percent** | **Cumulative Percent** |
| --- | --- | --- | --- | --- |
| Yes | 10 | 71.4 | 71.4 | 71.4 |
| No | 4 | 28.6 | 28.6 | 100.0 |
| **Total** | **14** | **100.0** | **100.0** |  |
