# Supplementary material for: Effectiveness of monitoring mechanisms in reducing immunization dropouts among infants in health facilities of Maridi County, South Sudan
Source: PLoS One. 2026 Sep 3;21(9):e0356268. doi: 10.1371/journal.pone.0356268 (PMC13541179; doi:10.1371/journal.pone.0356268)
Supplement: Supplementary file 9 [file pone.0356268.s009.docx]

**S9 Table. Frequency of quarterly performance review using the monitoring chart.**

Frequency and percentage distribution of how many times per quarter health facility staff check performance using the monitoring chart (N = 14).

**How many times in a quarter do you check performance using the monitoring chart?**

| **Response** | **Frequency** | **Percent** | **Valid Percent** | **Cumulative Percent** |
| --- | --- | --- | --- | --- |
| None | 1 | 7.1 | 7.1 | 7.1 |
| Three times | 8 | 57.1 | 57.1 | 64.3 |
| Two times | 2 | 14.3 | 14.3 | 78.6 |
| Once a quarter | 3 | 21.4 | 21.4 | 100.0 |
| **Total** | **14** | **100.0** | **100.0** |  |
