# Supplementary material for: Effectiveness of monitoring mechanisms in reducing immunization dropouts among infants in health facilities of Maridi County, South Sudan
Source: PLoS One. 2026 Sep 3;21(9):e0356268. doi: 10.1371/journal.pone.0356268 (PMC13541179; doi:10.1371/journal.pone.0356268)
Supplement: Supplementary file 10 [file pone.0356268.s010.docx]

**S10 Table. Correlation between monitoring chart updates and facility dropout rate.**

Spearman's rank correlation between facility dropout-rate classification and whether the monitoring chart was updated with the previous month's data (N = 14; rho = -0.725, p = .003).

**Correlations (Spearman's rho)**

|  |  | **Dropout rate of the health facility (classified)** | **Is the monitoring chart updated with previous month data** |
| --- | --- | --- | --- |
| Dropout rate of the health facility (classified) | Correlation coefficient | 1.000 | -.725** |
|  | Sig. (2-tailed) | . | .003 |
|  | N | 14 | 14 |
| Is the monitoring chart updated with previous month data | Correlation coefficient | -.725** | 1.000 |
|  | Sig. (2-tailed) | .003 | . |
|  | N | 14 | 14 |

*** Correlation is significant at the 0.01 level (2-tailed).*
