# Supplementary material for: Effectiveness of monitoring mechanisms in reducing immunization dropouts among infants in health facilities of Maridi County, South Sudan
Source: PLoS One. 2026 Sep 3;21(9):e0356268. doi: 10.1371/journal.pone.0356268 (PMC13541179; doi:10.1371/journal.pone.0356268)
Supplement: Supplementary file 11 [file pone.0356268.s011.docx]

**S11 Table. Accuracy of monitoring chart plotting, by facility dropout-rate category.**

Cross-tabulation of whether the monitoring chart was plotted correctly against facility dropout-rate category (no monitoring chart, <10%, >10%; N = 42).

**Was the monitoring chart plotted correctly? In relation to dropout rate**

| **Dropout rate** | **Response** | **Frequency** | **Percent** | **Valid Percent** | **Cumulative Percent** |
| --- | --- | --- | --- | --- | --- |
| No monitoring chart | No | 10 | 100.0 | 100.0 | 100.0 |
| Less than 10% | Yes | 23 | 95.8 | 95.8 | 95.8 |
|  | No | 1 | 4.2 | 4.2 | 100.0 |
|  | **Total** | **24** | **100.0** | **100.0** |  |
| More than 10% | Yes | 8 | 100.0 | 100.0 | 100.0 |
