# Supplementary material for: Effectiveness of monitoring mechanisms in reducing immunization dropouts among infants in health facilities of Maridi County, South Sudan
Source: PLoS One. 2026 Sep 3;21(9):e0356268. doi: 10.1371/journal.pone.0356268 (PMC13541179; doi:10.1371/journal.pone.0356268)
Supplement: Supplementary file 12 [file pone.0356268.s012.docx]

**S12 Table. Health facilities with boma health workers attached.**

Frequency and percentage distribution of health facilities reporting whether boma health workers (BHWs) are attached to the facility (N = 14).

**Does your health facility have boma health workers attached?**

| **Response** | **Frequency** | **Percent** | **Valid Percent** | **Cumulative Percent** |
| --- | --- | --- | --- | --- |
| BHWs exist | 9 | 64.3 | 64.3 | 64.3 |
| No BHWs | 5 | 35.7 | 35.7 | 100.0 |
| **Total** | **14** | **100.0** | **100.0** |  |
