# Supplementary material for: Effectiveness of monitoring mechanisms in reducing immunization dropouts among infants in health facilities of Maridi County, South Sudan
Source: PLoS One. 2026 Sep 3;21(9):e0356268. doi: 10.1371/journal.pone.0356268 (PMC13541179; doi:10.1371/journal.pone.0356268)
Supplement: Supplementary file 14 [file pone.0356268.s014.docx]

**S14 Table. Type of immunization referral received from boma health workers, by facility dropout-rate category.**

Cross-tabulation of the type of referral received (defaulters' referral, zero-dose referral, both, or none) against facility dropout-rate category (no monitoring chart, <10%, >10%; N = 14).

**What type of immunization referral do you receive from the boma health workers?**

| **Dropout rate** | **Response** | **Frequency** | **Percent** | **Valid Percent** | **Cumulative Percent** |
| --- | --- | --- | --- | --- | --- |
| No monitoring chart | Defaulters' referral | 1 | 33.3 | 33.3 | 33.3 |
|  | Zero-dose referral | 1 | 33.3 | 33.3 | 66.7 |
|  | 1 & 2 | 1 | 33.3 | 33.3 | 100.0 |
|  | **Total** | **3** | **100.0** | **100.0** |  |
| Less than 10% | Defaulters' referral | 6 | 85.7 | 85.7 | 85.7 |
|  | 1 & 2 | 1 | 14.3 | 14.3 | 100.0 |
|  | **Total** | **7** | **100.0** | **100.0** |  |
| More than 10% | Defaulters' referral | 2 | 50.0 | 50.0 | 50.0 |
|  | Zero-dose referral | 1 | 25.0 | 25.0 | 75.0 |
|  | No referrals | 1 | 25.0 | 25.0 | 100.0 |
|  | **Total** | **4** | **100.0** | **100.0** |  |

*"1 & 2" denotes facilities receiving both defaulters' referrals and zero-dose referrals.*
