# Supplementary material for: Effectiveness of monitoring mechanisms in reducing immunization dropouts among infants in health facilities of Maridi County, South Sudan
Source: PLoS One. 2026 Sep 3;21(9):e0356268. doi: 10.1371/journal.pone.0356268 (PMC13541179; doi:10.1371/journal.pone.0356268)
Supplement: Supplementary file 15 [file pone.0356268.s015.docx]

**S15 Table. Presence of boma health workers in relation to facility dropout rate.**

Cross-tabulation of whether boma health workers (BHWs) are present at the facility against facility dropout-rate category (no monitoring chart, <10%, >10%; N = 42).

**Presence of BHWs in relation to dropout rate**

| **Dropout rate** | **Response** | **Frequency** | **Percent** | **Valid Percent** | **Cumulative Percent** |
| --- | --- | --- | --- | --- | --- |
| No monitoring chart | No BHWs | 4 | 40.0 | 40.0 | 40.0 |
|  | BHWs exist | 6 | 60.0 | 60.0 | 100.0 |
|  | **Total** | **10** | **100.0** | **100.0** |  |
| Less than 10% | No BHWs | 2 | 8.3 | 8.3 | 8.3 |
|  | BHWs exist | 22 | 91.7 | 91.7 | 100.0 |
|  | **Total** | **24** | **100.0** | **100.0** |  |
| More than 10% | No BHWs | 5 | 62.5 | 62.5 | 62.5 |
|  | BHWs exist | 3 | 37.5 | 37.5 | 100.0 |
|  | **Total** | **8** | **100.0** | **100.0** |  |
