# Supplementary material for: Effectiveness of monitoring mechanisms in reducing immunization dropouts among infants in health facilities of Maridi County, South Sudan
Source: PLoS One. 2026 Sep 3;21(9):e0356268. doi: 10.1371/journal.pone.0356268 (PMC13541179; doi:10.1371/journal.pone.0356268)
Supplement: Supplementary file 17 [file pone.0356268.s017.docx]

**S17 Table. Correlation between health care worker defaulter tracing and facility dropout rate.**

Spearman's rank correlation between whether HCWs made an effort to trace identified defaulters and facility dropout rate (N = 42; rho = 0.354, p = .021).

**Correlations (Spearman's rho)**

|  |  | **Was there an effort by HCWs to link the identified defaulters and trace them** | **Dropout rate** |
| --- | --- | --- | --- |
| Was there an effort by HCWs to link the identified defaulters and trace them | Correlation coefficient | 1.000 | .354* |
|  | Sig. (2-tailed) | . | .021 |
|  | N | 42 | 42 |
| Dropout rate | Correlation coefficient | .354* | 1.000 |
|  | Sig. (2-tailed) | .021 | . |
|  | N | 42 | 42 |

** Correlation is significant at the 0.05 level (2-tailed).*
