# Supplementary material for: Effectiveness of monitoring mechanisms in reducing immunization dropouts among infants in health facilities of Maridi County, South Sudan
Source: PLoS One. 2026 Sep 3;21(9):e0356268. doi: 10.1371/journal.pone.0356268 (PMC13541179; doi:10.1371/journal.pone.0356268)
Supplement: Supplementary file 20 [file pone.0356268.s020.docx]

**S20 Table. Frequency of reviewing the tickler file for defaulter information.**

Frequency and percentage distribution of how often staff collect defaulter information from the tickler file (N = 14): none, daily, weekly, or monthly.

**When do you collect information about defaulters from the tickler file?**

| **Response** | **Frequency** | **Percent** | **Valid Percent** | **Cumulative Percent** |
| --- | --- | --- | --- | --- |
| None | 1 | 7.1 | 7.1 | 7.1 |
| Daily | 2 | 14.3 | 14.3 | 21.4 |
| Weekly | 6 | 42.9 | 42.9 | 64.3 |
| Monthly | 5 | 35.7 | 35.7 | 100.0 |
| **Total** | **14** | **100.0** | **100.0** |  |
