# Supplementary material for: Effectiveness of monitoring mechanisms in reducing immunization dropouts among infants in health facilities of Maridi County, South Sudan
Source: PLoS One. 2026 Sep 3;21(9):e0356268. doi: 10.1371/journal.pone.0356268 (PMC13541179; doi:10.1371/journal.pone.0356268)
Supplement: Supplementary file 21 [file pone.0356268.s021.docx]

**S21 Table. Perceived consequence of absent defaulter tracing systems on vaccine completion.**

Frequency and percentage distribution of respondent agreement with the statement that, absent a defaulter tracing system, children are less likely to complete vaccine doses such as penta-3 or measles (N = 14).

**When there is no defaulter tracing system in the health facility, many children will not complete their vaccine doses such as penta-3 or measles vaccine**

| **Response** | **Frequency** | **Percent** | **Valid Percent** | **Cumulative Percent** |
| --- | --- | --- | --- | --- |
| True | 10 | 71.4 | 71.4 | 71.4 |
| False | 4 | 28.6 | 28.6 | 100.0 |
| **Total** | **14** | **100.0** | **100.0** |  |
