# Supplementary material for: Effectiveness of monitoring mechanisms in reducing immunization dropouts among infants in health facilities of Maridi County, South Sudan
Source: PLoS One. 2026 Sep 3;21(9):e0356268. doi: 10.1371/journal.pone.0356268 (PMC13541179; doi:10.1371/journal.pone.0356268)
Supplement: Supplementary file 22 [file pone.0356268.s022.docx]

**S22 Table. Health facilities with documented immunization microplanning.**

Frequency and percentage distribution of whether the health facility has documented microplanning specifying outreach locations, stakeholders, a sketch map, and an analysis of the local immunization situation (N = 14).

**Does the health facility have documented microplanning specifying the location for outreach, stakeholders, sketch map, and analysed problem for the local situation on immunization services?**

| **Response** | **Frequency** | **Percent** | **Valid Percent** | **Cumulative Percent** |
| --- | --- | --- | --- | --- |
| Yes | 13 | 92.9 | 92.9 | 92.9 |
| No | 1 | 7.1 | 7.1 | 100.0 |
| **Total** | **14** | **100.0** | **100.0** |  |
