# Supplementary material for: Effectiveness of monitoring mechanisms in reducing immunization dropouts among infants in health facilities of Maridi County, South Sudan
Source: PLoS One. 2026 Sep 3;21(9):e0356268. doi: 10.1371/journal.pone.0356268 (PMC13541179; doi:10.1371/journal.pone.0356268)
Supplement: Supplementary file 23 [file pone.0356268.s023.docx]

**S23 Table. Recency of health facility microplanning updates.**

Frequency and percentage distribution of when the health facility's immunization microplanning was last updated: less than one year ago, one year ago, or 2–3 years ago (N = 14).

**When was the last time the health facility microplanning was updated or done?**

| **Response** | **Frequency** | **Percent** | **Valid Percent** | **Cumulative Percent** |
| --- | --- | --- | --- | --- |
| Less than a year | 12 | 85.7 | 85.7 | 85.7 |
| 1 year ago | 1 | 7.1 | 7.1 | 92.9 |
| 2–3 years ago | 1 | 7.1 | 7.1 | 100.0 |
| **Total** | **14** | **100.0** | **100.0** |  |
