# Supplementary material for: Effectiveness of monitoring mechanisms in reducing immunization dropouts among infants in health facilities of Maridi County, South Sudan
Source: PLoS One. 2026 Sep 3;21(9):e0356268. doi: 10.1371/journal.pone.0356268 (PMC13541179; doi:10.1371/journal.pone.0356268)
Supplement: Supplementary file 24 [file pone.0356268.s024.docx]

**S24 Table. One-way ANOVA of facility indicators across dropout-rate groups.**

One-way analysis of variance testing whether four facility-level indicators differ across dropout-rate group (no monitoring chart, <10%, >10%; N = 14): whether the monitoring chart is updated with the previous month's data, F(2,11) = 12.833, p = .001; whether the health facility has a defaulter tracing system, F(2,11) = 0.458, p = .644; whether immunization referrals were received from boma health workers or community mobilizers, F(2,11) = 3.929, p = .052; and the recency of the last microplanning update, F(2,11) = 4.939, p = .029.

| **ANOVA** | | | | | | |
| --- | --- | --- | --- | --- | --- | --- |
|  | | Sum of Squares | df | Mean Square | F | Sig. |
| Is the monitoring chart updated with previous month data | Between Groups | 2.000 | 2 | 1.000 | 12.833 | .001 |
|  | Within Groups | .857 | 11 | .078 |  |  |
|  | Total | 2.857 | 13 |  |  |  |
| Does the HF have defaulter tracing system | Between Groups | .071 | 2 | .036 | .458 | .644 |
|  | Within Groups | .857 | 11 | .078 |  |  |
|  | Total | .929 | 13 |  |  |  |
| Did you receive referral for immunization from the boma health workers or community mobilizers | Between Groups | .714 | 2 | .357 | 3.929 | .052 |
|  | Within Groups | 1.000 | 11 | .091 |  |  |
|  | Total | 1.714 | 13 |  |  |  |
| When was the last time the health facility microplanning was updated or done? | Between Groups | 4.190 | 2 | 2.095 | 4.939 | .029 |
|  | Within Groups | 4.667 | 11 | .424 |  |  |
|  | Total | 8.857 | 13 |  |  |  |
