# Supplementary material for: Effectiveness of monitoring mechanisms in reducing immunization dropouts among infants in health facilities of Maridi County, South Sudan
Source: PLoS One. 2026 Sep 3;21(9):e0356268. doi: 10.1371/journal.pone.0356268 (PMC13541179; doi:10.1371/journal.pone.0356268)
Supplement: Supplementary file 26 [file pone.0356268.s031.docx]

Reviewer #1 Comments on original manuscript, PLOS-25-26100

Several comments on the abstract:

Introduction – include more South Sudan-specific information.

Methods and conclusions – the study design cannot establish mechanisms of causality or assess effectiveness, which can only be done in randomized controlled trials. Who are the participants in the study? Vaccinators? How was the primary outcome defined? Stratified based on what? Further define what the exposed and unexposed groups of the cohort study are.

Results – no qualitative results were mentioned. Define BHWs. Unclear if dropout rates are measured per facility, per child, or per cohort.

Conclusion – The recommendation of scale-up is not supported by the data.

Introduction:
I recommend that the authors consult the STROBE checklist for cohort studies and include relevant items throughout their paper.

Authors should find more updated statistics about childhood immunization. The outdated 2019 statistics in the introduction should be replaced with more recent figures, as we know the COVID-19 pandemic reduced global childhood immunization coverage. Data from 2024 are available, so we would expect recent data to be incorporated in the introduction when referring to coverage and 0-dose children. For example, consider https://www.who.int/news-room/fact-sheets/detail/immunization-coverage that incorporates 2024 data. Additionally, authors cite an outdated version of IA2030 (the 2024 version is available). Moreover, 2024 estimates from South Sudan DTP1 coverage are available. Need references for immunization coverage rates.

The dropout rate is not defined. The dropout rate should have a clear operational definition that can be applied across settings.

Why do the authors switch from talking about immunization coverage to measles antigen performance without talking about measles coverage?

It’s unclear exactly what immunization monitoring mechanisms have worked or are being considered in the study. The specifics of what is being monitored, how, by whom, etc., need to be discussed. Also, what is the evidence of immunization monitoring for child vaccines? What has worked, and what are the gaps in evidence? Why have the authors focused on health facility microplanning and defaulter tracing systems? Authors should define what those mean.

Immunization dropout is defined as a child receiving at least one vaccine but not receiving subsequent vaccines. What about zero-dose vaccine children, who have never received immunizations? This is a common problem, which has gotten worse during and after the COVID-19 pandemic. How does this article address children who are unimmunized?

Authors cannot assess the effectiveness of any intervention with their study design. They are overstating their conclusions.
More details about the boma health initiative are warranted, such as who is involved, how it is implemented, and what is assessed.

Methods:

STROBE checklist 5 not clearly described (description of the setting, location, dates of exposure, dates of follow up and dates of data collection).

In addition, since this was also a qualitative study, I would like to see more details from the qualitative study reported based on the COREQ 32-item checklist of interviews and focus groups.

How were the 14 health facilities selected? Determining the sample size is not the same as selecting the health facilities.
Who are the participants?

STROBE checklist 7 and 8 have not been defined – we need to know all the outcomes, exposures, predictors, confounders, effective modifiers, and the sources of data and details of methods of measurement.

Show your sample size calculation. What assumptions were made?

Typo toasted

Since individuals from the same job often have different perspectives, I would have loved to see the authors interview all the vaccinators in the health facilities.

More details about the methodology of the qualitative study per the COREQ checklist. needs to be included. For example, items 9 through 23 of the checklist need to be addressed. In addition, how was the semi-structured interview guide developed?

What’s not clear is what the authors extracted and input into Excel and SPSS when this is largely a qualitative study.
The manuscript appears to conflate qualitative and quantitative analyses without clearly describing the methods used for each. It seems that this is a mixed-methods study, but that is not explicitly stated. The authors should clarify what quantitative analyses were conducted and specify what quantitative data were collected.

Author-state bivariable analysis was used to determine relationships, but that is not a statistical test. Was this logistical regression? Please expand on the statistical analysis for the quantitative part.

The authors report collecting data on immunization registers, health facilities, microplanning, and monitoring charts. However, the manuscript does not clearly describe how these variables were defined, operationalized, or measured. Given that these are central to the analysis, the authors should provide a detailed explanation of the data sources and collection methods for each variable to ensure clarity and reproducibility.

The authors should also specify the statistical measure used in these analyses (e.g., risk ratio or odds ratio), as this is not currently explained.

The manuscript does not provide sufficient detail on key data sources and tools. The authors should describe what the immunization registers contain and how information from them was used in the analysis. Similarly, the purpose and content of tickler files, defaulter tracing cards, and monitoring charts are not explained. Providing clear descriptions of these instruments is essential for readers to understand the data collection process and to assess the validity and replicability of the findings.

Results

The manuscript would benefit from the inclusion of tables or figures to summarize the data. For instance, demographic and facility characteristics could be presented in a descriptive table to enhance clarity. In addition, a table summarizing the quantitative regression analyses is expected.

The stated objective of the study is to ascertain the effectiveness of monitoring mechanisms in reducing immunization dropouts across health facilities. However, it is not clear how the presented analysis directly addresses or measures this objective. The association between the use of micro-monitoring tools and the updating of micro-planning documents, while potentially relevant, does not on its own demonstrate an effect on immunization dropout rates. The authors should clarify how this analysis contributes to assessing the effectiveness of monitoring mechanisms in reducing dropouts, and, if applicable, describe the analytical pathway linking these intermediate measures to the primary outcome.

The authors appear to include interpretive statements rather than presenting only the findings. For instance, explanations such as “accurate data capturing into the immunization register enhanced the completeness of the data tools” represent interpretation rather than results. Such explanatory or inferential statements would be more appropriately placed in the Discussion section, while the Results should focus on objectively reporting the observed data.

Based on the semi-structured interview data, the manuscript would benefit from a table summarizing the main themes accompanied by illustrative quotations to substantiate the findings. Similarly, a table summarizing the key results from the observational data is expected. The inclusion of these tables would enhance transparency, allow readers to assess the depth and credibility of the qualitative analysis, and improve the overall clarity of the results.

How did the authors address spillover in the locations between exposed and unexposed groups?

The manuscript should provide specific data on the actions taken by health facilities with desirable dropout rates to identify and trace defaulters. The current description of “effort” is vague and not operationally defined or measurable. The authors should clarify what specific activities or strategies were implemented and how these were assessed to allow for meaningful interpretation and comparison across facilities. The authors should define how they measured or categorized “effort.” For example: Low effort: No systematic review or tracing activities. Moderate effort: Passive record review without follow-up. High effort: Active tracing using multiple methods (register review, community outreach, phone calls).

Discussion
The study's novelty is not clearly established. The finding that monitoring immunization data can support tracking progress and reduce dropout rates is conceptually expected and has been well documented in prior literature. The authors should clarify what new insights their study provides beyond confirming that facilities that use monitoring tools perform better. For example, does the study offer a unique methodological approach, a deeper contextual understanding, or implementation insights that advance current knowledge? Clearly articulating the study’s added value would strengthen its contribution to the field.

The study needs to clarify its contribution beyond confirming that monitoring tools are associated with better immunization performance. The authors should explicitly articulate what is novel about their findings (e.g., a new implementation model, context-specific barriers, or unique operational lessons) and align their analytical approach with the stated objective of assessing effectiveness rather than mere association.

New findings are being added to the conclusion, including vaccine supply, which has not been mentioned in the discussion.

The authors are restating study results rather than interpreting them within a broader conceptual or theoretical context. The authors do not critically examine why or how monitoring mechanisms influenced dropout rates, nor do they acknowledge the methodological limitations of their study. As written, the discussion reads as confirmatory and, at times, overstates the study’s conclusions about effectiveness, which can be measured in an RCT. The authors should strengthen this section by providing a more analytical interpretation of findings, situating them within existing literature on immunization system performance, and explicitly addressing the study’s limitations and implications for policy and practice.
Although the authors include brief recommendations directed at policymakers within the conclusion, these points are not clearly separated or developed into a distinct section. The authors should consider presenting a concise “Implications for Policymakers” section that translates their findings into actionable guidance.
