# Supplementary material for: Effectiveness of monitoring mechanisms in reducing immunization dropouts among infants in health facilities of Maridi County, South Sudan
Source: PLoS One. 2026 Sep 3;21(9):e0356268. doi: 10.1371/journal.pone.0356268 (PMC13541179; doi:10.1371/journal.pone.0356268)
Supplement: Supplementary file 27 [file pone.0356268.s029.docx]

Academic Editor Comments

DETAILED COMMENTS (most minor)

Abstract-Intro

‘coverage reduced to 84%’. At what time point?

‘Despite the efforts employed to improve the immunization coverage, SUBJECT NEEDED HERE remained below 85%.’

Abstract—methods/results

‘BHWs’—make sure the abbreviations are written out first time they are used.

Explain in abstract (briefly) what a Boma Health Worker is, also state how long the study lasted (i.e. over what period of time did changes occur?)

Abstract-Conclusions

Can you distinguish between the monitoring tools used by internal health workers and the external monitoring and the effect of BHWs?

Introduction

‘The number of infants vaccinated globally reduced, specifically, uptake of 3rd dose of pentavalent.’ Statements like this need to have a time frame. The next sentence is not clear.

‘The study intends to determine the use of immunization monitoring mechanism as a strategy to improve the county’s performance of 3rd dose of pentavalent, according to 2022 data, which is 82% and the quite low measles vaccine coverage at 55% to the minimum target of 85% through the proper use of immunization monitoring mechanisms. ‘ This sentence is repetitive about using monitoring mechanisms.

Why ‘Nevertheless’? seems like it’s all saying the same thing.

‘monthly goals on a timely fashion, provide basis for ….’ should be ‘in’ a timely fashion and ‘provideS’ and ‘reflecting’

‘vaccinator was selected based on toasted coin’ should be based on a tossed coin or based on a coin toss (spell check helping you too much here!)

‘had a written informed consent, that’ (no comma should be here between subject and verb)

‘This examined whether the presence of BHWs in health facilities was either attributed to having low dropout rates (less than 10%) or high dropout rates (more than 10%).’ Perhaps rather than ‘attributed to’, you meant to say ‘associated with’. The sentence now seems to say that the dropout rate caused the BHW to be in the health facility. That could happen, if authorities sent BHWs based on dropout rate, but I think you mean that the BHW influenced the dropout rate.

‘The researcher provided written informed consent to the participants,’. It seems that the researcher OBTAINED written informed consent FROM the participants. What was the original language of the IC? **Please state whether the IC translation was written (and verified in some way and approved by ethics committee) or by a script that was oral (approved?), or whether it was done each time verbally by the person obtaining informed consent.**

**RESULTS**

**‘..**Out of the 14 health facilities, (12, 86%) were supported… ‘ The 12 should before parentheses. Same for the 2 later on in the sentence, etc etc.

**‘**Majority of the male respondents (11, 79%)….’ Be clear that you mean by respondent the person responding on behalf of the health facility.

The % of males who had secondary education seems to be 100%. The % of all respondents who had secondary education is 79%. Does this mean there were just 3 women responding? Make that respondentS.

‘Use of Immunization monitoring chart to tract immunization performance.’ Should be TRACK

‘(1, 7%) used monitoring chart to realize their defaulters.’ Should be ‘recognize’ or ‘identify’, not realize.

Are these methods of identifying defaulters overlapping (more than one used) or exclusive categories (either/or)?

**What does ‘the observation data’ mean? For the discussion, how credible are these data? Were people trying extra hard when observers were there? Was there another way to validate the data?**

**‘…** was statistically significant (Mean difference: 1.78, 95% CI: 1.22 – 2.35) in reducing defaulters in the health facilities.’ Use of monitoring tools was ASSOCIATED with a lower rate of defaulters. This does not prove it was the cause, although that may well be true (to talk about in the Discussion).

‘health facility microplanning document been updated,’ should say ‘being updated’. Or ‘that had been updated’.

‘As much as monitoring chart is a performance tool, it should be used to display the performance and gauge the success of immunization rather than identifying the immunization defaulters.’’. This sentence expresses a general opinion and should be in the Discussion section, not results.

Presence of BHWs in the health facilities is a key factor…. This paragraph would be well illustrated by a table to be refo ed tp in the text

‘Presence of BHWs attached to the health facilities should facilitate referrals of the defaulters after the health worker had identified and hand over a list of defaulters who should be found in the community and referred to complete their vaccination schedule. The health workers felt motivated to support the tracing the defaulters as BHWs supported them in ensuring that…… ‘This belongs in the discussion.

**‘**had fifty percent (50%) effort to identify the defaulters and trace them.’. Does this mean 50% of the centers attempted to identify defaulters? Or that all of them tried half as hard?

‘Ninety three percent (13, 93%) of the respondents confirmed that their facilities have defaulter tracing system,…’ These data were also mentioned earlier, but it is much clearer in this paragraph. Suggest eliminating the duplication and showing this in a table, referred to in text.

Disc

the more health facilities have monitoring charts, the lesser they ‘ would have dropouts’, Agree with the sentiment here, but it is an association, not proven (suggests that) and also not written clearly. They do or not, yes or no, correct? But this is written as if each facility can have more or less of a chart.

‘The study finding agreed….’ The study has findingS

‘A relationship existed between health workers’ effort to identify defaulters, link them for immunization service and reduced dropout rate. This indicated the more health workers put effort to identify the defaulters to be linked for immunization service, it reduces the dropout rate by 66%.’ How did you identify the amount of effort each health worker put into the identification of defaulters. I didn’t see quantitative data, which would be hard or impossible to get. So this statement seems to overinterpret your results. It is probably true, but did you measure the amount of effort?

A good way to express some of these thoughts might be to say ‘our data are consistent with the findings of so-and-so, who showed….’.

In general, the discussion could be a bit better organized. It seems repetitive.

Conclusion:

How does lack of vaccine supply fit into this? Are vaccines ordered based on the records? Are people whose children missed vaccination because of short supply then recalled when the vaccine comes in?

‘	Monitoring charts and defaulter tracing system were less commonly used. Gaps of no monitoring chart in other health facilities were attributed to poor performance in monitoring of immunization services.’ A general problem in understanding this is that monitoring seems to mean use of the charts sometimes, but it really doesn’t. So what IS monitoring and who does it? This could be better explained in the introduction/methods.
