# Supplementary material for: Effectiveness of monitoring mechanisms in reducing immunization dropouts among infants in health facilities of Maridi County, South Sudan
Source: PLoS One. 2026 Sep 3;21(9):e0356268. doi: 10.1371/journal.pone.0356268 (PMC13541179; doi:10.1371/journal.pone.0356268)
Supplement: Supplementary file 28 [file pone.0356268.s032.docx]

**Response to Academic Editor Comments**

**Manuscript title:** *Effectiveness of Monitoring Mechanisms in Reducing Immunization Dropouts Among Infants in Health Facilities of Maridi County, South Sudan*

**Journal:** PLOS ONE

**Manuscript number:** PONE-D-25-26100

**Authors:** Daniel Opinile Mark Igga, Nzomo Mwita, Kennedy Muni

Dear Editor,

We sincerely thank the Academic Editor for the careful and constructive review of our manuscript. The comments identified genuine weaknesses in clarity, precision, and organization that we believe were important to address. We have revised the manuscript thoroughly in response to every point raised. Below we provide a point-by-point response to each comment. For ease of reference, each editor comment is quoted verbatim (shaded grey), followed by our response and a description of the specific change made in the revised manuscript. Passages added or substantially rewritten in the manuscript are indicated in the third column.

We hope the revisions satisfy the editor’s concerns and that the manuscript is now suitable for publication in PLOS ONE.

Yours sincerely,

Daniel Opinile Mark Igga (on behalf of all authors)

**Section 1: Abstract**

**Comment 1** — Abstract – Introduction

| **Editor Comment** | **Authors’ Response** | **Manuscript Change** |
| --- | --- | --- |
| "coverage reduced to 84%". At what time point? | The editor is correct that the time point was missing, which made the statement imprecise and unverifiable. Global third-dose pentavalent coverage was 86% in 2019, declined to 84% in 2020, and fell further to 82% in 2021 — a three-year trend reported by the Immunization Agenda 2030. Specifying these years is essential for readers to contextualise the data against global benchmarks. | Revised abstract (Introduction): "Third-dose pentavalent vaccine coverage stood at 86% globally in 2019, declining to 84% in 2020 and further to 82% in 2021, remaining below the 85% minimum target set by the Immunization Agenda 2030." All three years are now explicit. |

**Comment 2** — Abstract – Introduction

| **Editor Comment** | **Authors’ Response** | **Manuscript Change** |
| --- | --- | --- |
| "Despite the efforts employed to improve the immunization coverage, SUBJECT NEEDED HERE remained below 85%." — A subject is missing from this sentence. | We agree. The sentence was grammatically incomplete. The intended subject was "immunization coverage," which had been dropped in editing. The sentence has been rewritten to remove the dangling clause entirely and incorporate the substance into a fuller, subject-complete sentence. | Revised abstract (Introduction): The sentence now reads: "...declining to 84% in 2020 and further to 82% in 2021, consistently falling short of the 85% floor target set by the Immunization Agenda 2030." The incomplete clause has been eliminated. |

**Comment 3** — Abstract – Methods/Results

| **Editor Comment** | **Authors’ Response** | **Manuscript Change** |
| --- | --- | --- |
| "BHWs" — make sure the abbreviations are written out first time they are used. | We agree. The abbreviation "BHW" appeared in the abstract without prior definition, which is inconsistent with PLOS ONE style and would confuse readers unfamiliar with South Sudan's community health architecture. The term is now spelled out in full on first use in the abstract, with the abbreviation provided in parentheses immediately afterward. | Revised abstract (Results): "...Boma Health Workers (BHWs) — community-level health personnel who mobilize, trace, and refer immunization defaulters — were attached to 64% of facilities." Subsequent uses of "BHW/BHWs" throughout the abstract and manuscript are abbreviated. |

**Comment 4** — Abstract – Methods/Results

| **Editor Comment** | **Authors’ Response** | **Manuscript Change** |
| --- | --- | --- |
| Explain in abstract (briefly) what a Boma Health Worker is, also state how long the study lasted (i.e. over what period of time did changes occur?). | Both points are well taken. First, the Boma Health Worker is a programme-specific cadre unique to South Sudan's health system, and readers outside the country would have no frame of reference for the term without a brief explanation. Second, without a time frame readers cannot judge how meaningful or durable the observed dropout reductions are. We have addressed both. | Revised abstract (Methods): The study duration is now explicit: "...conducted from 1 October to 31 December 2023."  Revised abstract (Results): BHWs are defined inline: "Boma Health Workers (BHWs) — community-level health personnel who mobilize, trace, and refer immunization defaulters..." |

**Comment 5** — Abstract – Conclusions

| **Editor Comment** | **Authors’ Response** | **Manuscript Change** |
| --- | --- | --- |
| Can you distinguish between the monitoring tools used by internal health workers and the external monitoring and the effect of BHWs? | This is an important conceptual distinction that the original abstract conflated. The monitoring chart, immunization register, and defaulter tracing system are facility-based (internal) tools used by vaccinators and health workers to track coverage and identify individual defaulters. BHWs operate at the community (external) level, mobilizing households, tracing missing children, and referring them back to facilities. The abstract conclusions have been rewritten to make this distinction explicit, so readers understand that the two mechanisms are complementary rather than interchangeable. | Revised abstract (Conclusions): "Both facility-based tools (monitoring charts, defaulter tracing systems) and community-based mechanisms (BHWs) are independently associated with lower immunization dropout rates, and their combination appears to have a complementary effect." The distinction between internal and external monitoring is now explicit in the conclusions. |

**Section 2: Introduction**

**Comment 6** — Introduction

| **Editor Comment** | **Authors’ Response** | **Manuscript Change** |
| --- | --- | --- |
| "The number of infants vaccinated globally reduced, specifically, uptake of 3rd dose of pentavalent." Statements like this need to have a time frame. The next sentence is not clear. | We agree on both counts. The original statement was both imprecise (no year specified) and syntactically awkward ("specifically, uptake of 3rd dose of pentavalent" does not complete a grammatical thought). The subsequent sentence about the Immunization Agenda 2030 target was also unclear in its relationship to the preceding figures. We have completely rewritten this passage with explicit years (2019, 2020, 2021) drawn from the Immunization Agenda 2030 global data, and the relationship between the observed decline and the target threshold is now stated directly. | Revised Introduction: "Third-dose pentavalent coverage, a widely used proxy for immunization system performance, stood at 86% globally in 2019 but slipped to 84% in 2020 and 82% in 2021, consistently falling short of the 85% floor target set by the Immunization Agenda 2030." The unclear follow-on sentence has been removed and replaced with a direct statement about what falling short of the target means for program planning. |

**Comment 7** — Introduction

| **Editor Comment** | **Authors’ Response** | **Manuscript Change** |
| --- | --- | --- |
| "The study intends to determine the use of immunization monitoring mechanism as a strategy to improve the county's performance of 3rd dose of pentavalent, according to 2022 data, which is 82% and the quite low measles vaccine coverage at 55% to the minimum target of 85% through the proper use of immunization monitoring mechanisms." This sentence is repetitive about using monitoring mechanisms. | We agree that the phrase "immunization monitoring mechanism" appeared twice in the same sentence and the sentence was also structurally unwieldy. The entire passage has been reorganised. The Maridi County coverage figures (82% pentavalent-3 and 55% measles in 2022) now appear in a standalone sentence providing factual context, and the study aim is stated separately and more concisely. | Revised Introduction: The county's coverage gap is stated as: "...2022 programme data revealed 82% third-dose pentavalent coverage and 55% measles vaccine coverage in the county — both below the national 85% target." The study purpose is then stated separately without repetition of the phrase "monitoring mechanisms." |

**Comment 8** — Introduction

| **Editor Comment** | **Authors’ Response** | **Manuscript Change** |
| --- | --- | --- |
| Why "Nevertheless"? It seems like it's all saying the same thing. | The editor is right. "Nevertheless" implies a contrast or concession, but the surrounding sentences were building the same argument rather than pivoting to a contrasting point. The word was being used as a filler transition. The paragraph has been restructured so that each sentence advances a distinct idea: (1) the coverage gap in Maridi County, (2) what the target is, (3) why monitoring and community tracing matter, and (4) the study aim. The transitional word "Nevertheless" has been removed entirely. | Revised Introduction: The paragraph has been restructured. "Nevertheless" has been deleted, and the sentences that followed it have been reorganised to flow logically without a false contrast marker. |

**Comment 9** — Introduction

| **Editor Comment** | **Authors’ Response** | **Manuscript Change** |
| --- | --- | --- |
| "monthly goals on a timely fashion, provide basis for…" should be "in a timely fashion" and "provides" and "reflecting". | We thank the editor for catching these grammatical errors. "On a timely fashion" is incorrect idiom; the correct phrase is "in a timely fashion." The verb "provide" should agree with its singular subject ("this tool") and read "provides." "Reflect" in the participial phrase should read "reflecting" for grammatical parallelism. All three corrections have been made. | Revised Introduction: The sentence describing the monitoring chart now reads: "...set and evaluate monthly goals in a timely fashion, provides a basis for comparing performance across time periods...and reflects the situation accurately." All three grammatical corrections are incorporated. |

**Section 3: Materials and Methods**

**Comment 10** — Methods

| **Editor Comment** | **Authors’ Response** | **Manuscript Change** |
| --- | --- | --- |
| "vaccinator was selected based on toasted coin" should be based on a tossed coin or based on a coin toss. | This is a clear spell-checker substitution error ("toasted" for "tossed") that we missed in proofreading. We apologize for the oversight. The phrase has been corrected throughout the manuscript. | Revised Methods (Sampling): "...one was enrolled in the study by coin toss, yielding 13 participants per stratum (26 total)." All instances of "toasted coin" have been replaced with "coin toss." |

**Comment 11** — Methods

| **Editor Comment** | **Authors’ Response** | **Manuscript Change** |
| --- | --- | --- |
| "had a written informed consent, that" — no comma should be here between subject and verb. | Correct. The comma between "consent" and "that" incorrectly separates the noun phrase from its relative clause. The comma has been removed. The sentence has also been further revised for clarity (see Comment 12 below). | Revised Methods (Data Collection): The comma has been removed. The sentence now reads: "...a semi-structured interview guide that included a written informed consent section..." |

**Comment 12** — Methods

| **Editor Comment** | **Authors’ Response** | **Manuscript Change** |
| --- | --- | --- |
| "This examined whether the presence of BHWs in health facilities was either attributed to having low dropout rates (less than 10%) or high dropout rates (more than 10%)." Perhaps rather than "attributed to," you meant to say "associated with." The sentence now seems to say that the dropout rate caused the BHW to be in the health facility. | This is an important clarification. The original phrasing was causally ambiguous — it could imply that authorities deployed BHWs to facilities because those facilities already had high dropout rates (i.e., dropout rate caused BHW presence), when in fact we were testing the reverse hypothesis (BHW presence influences dropout rate). The correct statistical framing is one of association, not attribution. We have replaced "attributed to" with "associated with" throughout the manuscript and added a brief note in the Discussion clarifying that the study design does not permit causal inference. | Revised Methods (Analysis): "Chi-square tests were used to examine whether BHW presence in health facilities was associated with lower dropout rates (below 10%) or higher dropout rates (above 10%)." The word "attributed" has been replaced with "associated" throughout. |

**Comment 13** — Methods

| **Editor Comment** | **Authors’ Response** | **Manuscript Change** |
| --- | --- | --- |
| "The researcher provided written informed consent to the participants." It seems that the researcher OBTAINED written informed consent FROM the participants. What was the original language of the informed consent? Please state whether the IC translation was written (and verified in some way and approved by ethics committee) or by a script that was oral (approved?), or whether it was done each time verbally by the person obtaining informed consent. | The editor is entirely correct, and we apologize for the imprecise language. The researcher obtained written informed consent from the participants — not the other way around. Additionally, we recognize that the original manuscript failed to specify the informed consent language protocol, which is an important omission for a study conducted in a multilingual, low-resource setting.  To clarify: The informed consent form was prepared in English. Because many participants are more comfortable in local languages, the researcher verbally interpreted the consent form into the participant's preferred language — Juba Arabic, Muru, Azande, Baka, or Avokaya — prior to the participant signing. This verbal translation protocol was reviewed and approved by the AMIU Ethics and Scientific Review Committee and the South Sudan MOH Research Ethics Review Board as part of the study ethics application. Consent was obtained in writing from every participant; the signature confirmed that the verbal explanation had been understood and that the participant agreed voluntarily to take part. | Revised Methods (Ethics): "Written informed consent was obtained from every enrolled vaccinator before data collection began. The consent form was prepared in English and verbally interpreted by the researcher into each participant's preferred language (Juba Arabic, Muru, Azande, Baka, or Avokaya) prior to signature. This verbal interpretation protocol was reviewed and approved as part of the study's ethics application to the AMIU ESRC and the South Sudan MOH-RERB." |

**Section 4: Results**

**Comment 14** — Results – Formatting

| **Editor Comment** | **Authors’ Response** | **Manuscript Change** |
| --- | --- | --- |
| "Out of the 14 health facilities, (12, 86%) were supported…" The 12 should be before parentheses. Same for the 2 later on in the sentence, etc. | We agree. Placing the count outside the parentheses and the percentage inside is the correct and widely adopted convention in biomedical writing — e.g., "12 (86%)" rather than "(12, 86%)". This formatting error appeared throughout the original Results section. All instances have been corrected. | Revised Results: All frequency-percentage pairs are now formatted as "n (%)" throughout — e.g., "12 (86%)," "3 (21%)," "13 (93%)" — consistent with PLOS ONE style. |

**Comment 15** — Results – Demographics

| **Editor Comment** | **Authors’ Response** | **Manuscript Change** |
| --- | --- | --- |
| "Majority of the male respondents (11, 79%)…" Be clear that you mean by respondent the person responding on behalf of the health facility. | We agree that the term "respondent" was ambiguous and could be read as referring to a member of the general public. Since each health facility contributed one vaccinator to the study, the "respondent" is always the vaccinator selected to represent that facility. We have clarified this at first use and maintained the clarification consistently throughout the Results section. | Revised Results (Demographics): "...14 respondents (one vaccinator per facility, selected to represent their health facility)." Subsequent mentions describe respondents as "vaccinators" or "health facility representatives" to avoid ambiguity. |

**Comment 16** — Results – Demographics

| **Editor Comment** | **Authors’ Response** | **Manuscript Change** |
| --- | --- | --- |
| The % of males who had secondary education seems to be 100%. The % of all respondents who had secondary education is 79%. Does this mean there were just 3 women responding? Make that respondentS. | The editor is correct on both counts. All 11 male respondents attained secondary education (100% of males), while all 3 female respondents attained only primary education — meaning the 3 women were indeed the entire female sample. We have made this explicit in the text to avoid misinterpretation and have used the plural "respondents" consistently. We have also presented these figures in a table (Table 1 in the revised manuscript) to make the breakdown immediately transparent. | Revised Results (Demographics): "When disaggregated by sex, all 11 male respondents had attained secondary-level education, while all three female respondents had attained primary-level education only." Table 1 presents the full demographic breakdown with counts and percentages. "Respondents" (plural) is used throughout. |

**Comment 17** — Results – Monitoring Chart

| **Editor Comment** | **Authors’ Response** | **Manuscript Change** |
| --- | --- | --- |
| "Use of Immunization monitoring chart to tract immunization performance." Should be TRACK. | Correct — "tract" is a noun, not a verb. The intended word is the verb "track." This typographical error has been corrected throughout the manuscript. | Revised Results (sub-section heading and body text): "track" replaces "tract" in all instances. |

**Comment 18** — Results – Monitoring Chart

| **Editor Comment** | **Authors’ Response** | **Manuscript Change** |
| --- | --- | --- |
| "(1, 7%) used monitoring chart to realize their defaulters." Should be "recognize" or "identify," not realize. | Agreed. "Realize" does not convey the intended meaning in this context. The intended meaning is that health workers used the monitoring chart to identify or recognize which infants had missed their scheduled vaccination. "Identify" is the more precise and standard term in public health writing and has been used throughout the revised manuscript. | Revised Results: "...1 (7%) used the monitoring chart itself to identify their defaulters." "Realize" has been replaced by "identify" throughout. |

**Comment 19** — Results – Monitoring Chart

| **Editor Comment** | **Authors’ Response** | **Manuscript Change** |
| --- | --- | --- |
| Are these methods of identifying defaulters overlapping (more than one used) or exclusive categories (either/or)? | This is an important methodological clarification. The categories were not mutually exclusive — several facilities reported using more than one tool to identify defaulters (e.g., both immunization registers and tickler files). The original text implied exclusivity by presenting the percentages without qualification. We have corrected this by adding an explicit clarification that respondents could report more than one method, and we note that the percentages therefore do not sum to 100%. | Revised Results: "Respondents were not limited to a single answer; several facilities used more than one tool. Among all responses, 7 (50%) cited immunization registers as their primary approach, 4 (29%) used tickler files, 2 (14%) relied on dedicated defaulter tracing cards, and 1 (7%) used the monitoring chart itself. These categories are not mutually exclusive." |

**Comment 20** — Results – Observation Data Credibility

| **Editor Comment** | **Authors’ Response** | **Manuscript Change** |
| --- | --- | --- |
| What does "the observation data" mean? For the discussion, how credible are these data? Were people trying extra hard when observers were there? Was there another way to validate the data? | This is an important question that touches on observer bias (the Hawthorne effect) and data validity. "Observation data" referred to data collected using the monthly observation checklist completed by the researcher during facility visits, as opposed to the self-reported interview data. We have clarified the terminology throughout.  Regarding credibility: we acknowledge that health workers may have performed better (or appeared to perform better) during months when they knew the researcher was visiting, which is a recognized limitation of observational research using checklists. We have added an explicit discussion of this limitation in the Limitations paragraph. In terms of validation: the observation checklist drew primarily on existing facility records — monthly summary reports, immunization registers, and monitoring charts — rather than on real-time observation of health worker behavior. This partially mitigates the Hawthorne effect, because the records reflect cumulative monthly performance rather than behavior at a single point in time. We did not conduct a separate independent validation exercise, and this is acknowledged as a study limitation. | Revised Methods: The observation checklist is now described clearly: "The observation checklist...drew on existing facility records (monthly summary reports, immunization registers, monitoring charts) rather than on real-time behavioral observation, which partially mitigates observer-induced performance effects."  Revised Limitations: "The observation checklist was completed during researcher visits; health workers may have performed better during observed months than at other times, potentially inflating the apparent performance of facilities with weaker routine practices." |

**Comment 21** — Results – Causal Language

| **Editor Comment** | **Authors’ Response** | **Manuscript Change** |
| --- | --- | --- |
| "was statistically significant (Mean difference: 1.78, 95% CI: 1.22–2.35) in reducing defaulters in the health facilities." Use of monitoring tools was ASSOCIATED with a lower rate of defaulters. This does not prove it was the cause. | The editor is absolutely right. In an observational study without randomization, statistical significance establishes association, not causation. Claiming that monitoring tool use "reduced" defaulters implies a causal mechanism that our design cannot support. We have revised all such language throughout the manuscript to use the appropriate associative framing ("was associated with," "was linked to," "corresponds to"), and we have added a statement in the Discussion explicitly noting this limitation of the study design. | Revised Results: "Use of monitoring tools was significantly associated with lower defaulter rates (mean difference: 1.79, 95% CI: 1.22–2.35, p < 0.001)." The word "reducing" has been replaced throughout with association language. A note in the Limitations paragraph states: "Because facilities were not randomised to intervention conditions, it is not possible to attribute differences in dropout rates causally to the monitoring mechanisms under study." |

**Comment 22** — Results – Grammar

| **Editor Comment** | **Authors’ Response** | **Manuscript Change** |
| --- | --- | --- |
| "health facility microplanning document been updated," should say "being updated" or "that had been updated." | Agreed. "Been updated" is grammatically incomplete without a preceding auxiliary verb. We have revised the sentence to use the relative clause form "that had been updated," which is grammatically correct and clearly conveys the intended meaning. | Revised Results: "...use of the monitoring chart was associated with the health facility microplanning document having been recently updated, with a correlation coefficient of 54% and p = 0.04." |

**Comment 23** — Results – Opinion in Results Section

| **Editor Comment** | **Authors’ Response** | **Manuscript Change** |
| --- | --- | --- |
| "As much as monitoring chart is a performance tool, it should be used to display the performance and gauge the success of immunization rather than identifying the immunization defaulters." This sentence expresses a general opinion and should be in the Discussion section, not results. | The editor is correct. This sentence represents the authors' interpretation and normative judgment about how the monitoring chart should be used, which is not a finding but an opinion derived from the findings. Opinions and interpretations belong in the Discussion section. The sentence has been removed from the Results and a refined version, grounded in the data and citing relevant evidence, has been incorporated into the Discussion section where it belongs. | Removed from Results. A related interpretive point is now made in the Discussion: "Most respondents correctly relied on immunization registers and defaulter tracing cards — rather than the monitoring chart itself — to identify individual defaulters, reflecting appropriate understanding of each tool's purpose. The monitoring chart is most useful for tracking aggregate coverage trends and triggering corrective action, while registers and defaulter cards are the operational tools for identifying and following up individual children." |

**Comment 24** — Results – BHW Paragraph

| **Editor Comment** | **Authors’ Response** | **Manuscript Change** |
| --- | --- | --- |
| "Presence of BHWs in the health facilities is a key factor…" This paragraph would be well illustrated by a table to be referred to in the text. | We agree completely. Presenting the BHW presence data alongside dropout rate categories in prose alone makes the pattern difficult to see. We have added Table 3 in the revised manuscript, which cross-tabulates BHW attachment status (BHW present vs. no BHW) against dropout rate category (< 10%, > 10%, no monitoring chart) across the 42 facility-month observations. The table is cited in the text at the appropriate point. | Revised Results: Table 3 ("BHW attachment status in relation to dropout rate category across 42 facility-month observations") has been added and is referenced in the text: "Table 3 presents the relationship between BHW presence and dropout rate category." |

**Comment 25** — Results – Misplaced Interpretive Sentences

| **Editor Comment** | **Authors’ Response** | **Manuscript Change** |
| --- | --- | --- |
| "Presence of BHWs attached to the health facilities should facilitate referrals of the defaulters after the health worker had identified and hand over a list of defaulters…The health workers felt motivated to support the tracing the defaulters as BHWs supported them…" This belongs in the discussion. | We agree. These two sentences describe a proposed operational mechanism and a subjective experience reported by health workers, both of which are interpretive and explanatory in nature rather than factual findings. They have been removed from the Results section and incorporated into the Discussion, where they appropriately inform the interpretation of the BHW association data. | Removed from Results. Moved to Discussion: "...when health workers take the initiative to compile lists of defaulters, BHWs have clearer targets to follow up. This bidirectional dynamic suggests that strengthening either side of the community-facility interface independently may yield diminishing returns." |

**Comment 26** — Results – Ambiguous "50% effort"

| **Editor Comment** | **Authors’ Response** | **Manuscript Change** |
| --- | --- | --- |
| "had fifty percent (50%) effort to identify the defaulters and trace them." Does this mean 50% of the centers attempted to identify defaulters? Or that all of them tried half as hard? | This sentence was indeed ambiguous between two very different interpretations. The intended meaning was that, among facilities with a dropout rate above 10%, health workers made active, documented efforts to identify and link defaulters in only 50% of the facility-month observation cycles — meaning that in the other 50% of observations at those facilities, no such effort was recorded. This is a binary (effort/no effort) observation recorded in the checklist, not a measure of intensity of effort. We have rewritten the sentence to make this clear. | Revised Results: "Among facilities with dropout rates above 10%, active and documented efforts by health workers to identify and link defaulters were recorded in only 50% of facility-month observations — meaning that in the remaining observations, no such effort was documented." The ambiguous phrase "50% effort" has been eliminated. |

**Comment 27** — Results – Duplication

| **Editor Comment** | **Authors’ Response** | **Manuscript Change** |
| --- | --- | --- |
| "Ninety three percent (13, 93%) of the respondents confirmed that their facilities have defaulter tracing system…" These data were also mentioned earlier, but it is much clearer in this paragraph. Suggest eliminating the duplication and showing this in a table, referred to in text. | Agreed. The defaulter tracing system data appeared twice in the Results section — once in summary form and again in a later paragraph with more detail. This created unnecessary repetition and made it harder for readers to locate the primary presentation of these findings. We have consolidated all defaulter tracing system data into a single presentation (Table 4 in the revised manuscript) and removed the earlier duplicated reference. | Revised Results: Table 4 ("Defaulter tracing system characteristics and related practices, N = 14 facilities") consolidates all defaulter tracing data including: proportion with a tracing system, card-filling rate, tickler file knowledge, stockout recognition, and HFMP update status. Earlier duplicated text has been removed. |

**Section 5: Discussion**

**Comment 28** — Discussion – Association vs. Causation

| **Editor Comment** | **Authors’ Response** | **Manuscript Change** |
| --- | --- | --- |
| "the more health facilities have monitoring charts, the lesser they would have dropouts." Agree with the sentiment here, but it is an association, not proven, and also not written clearly. They do or not, yes or no, correct? But this is written as if each facility can have more or less of a chart. | The editor raises two valid points. First, the causal language problem: the statement implies a proven mechanism, when in fact the study establishes an association. Second, the comparative phrasing ("the more...the lesser") implies a continuous variable (degree of chart-having), when in reality monitoring chart presence is binary — a facility either has one or does not. Both errors have been corrected. | Revised Discussion: "Facilities that had a monitoring chart, and particularly those that kept it updated monthly, were significantly less likely to record a dropout rate above 10% during the study period (Spearman r = −0.725, p = 0.003). Our data are consistent with growing evidence that the regular use — rather than mere availability — of routine immunization data is what drives program improvement." |

**Comment 29** — Discussion – Grammar

| **Editor Comment** | **Authors’ Response** | **Manuscript Change** |
| --- | --- | --- |
| "The study finding agreed…" The study has findingS. | Correct. "Finding" in the singular implies a single result, whereas the study produced multiple findings. The plural "findings" should be used. This has been corrected. | Revised Discussion: "The study findings are consistent with..." and similar formulations are used throughout. All singular instances of "finding" where the plural is meant have been corrected. |

**Comment 30** — Discussion – Overinterpretation of Effort

| **Editor Comment** | **Authors’ Response** | **Manuscript Change** |
| --- | --- | --- |
| "A relationship existed between health workers' effort to identify defaulters, link them for immunization service and reduced dropout rate. This indicated the more health workers put effort to identify the defaulters to be linked for immunization service, it reduces the dropout rate by 66%." How did you identify the amount of effort each health worker put into the identification of defaulters? I didn't see quantitative data, which would be hard or impossible to get. So this statement seems to overinterpret your results. | The editor has identified a genuine overinterpretation. "Effort" as used in the original manuscript referred to a binary observation recorded in the monthly checklist — whether the health worker made any documented attempt (yes/no) to identify and link defaulters during that month, not a quantified measure of effort intensity. The Spearman correlation coefficient of 0.354 (p = 0.021) reflects the association between this binary indicator and the dropout rate category, not a measure of how hard health workers tried. Describing this as a "66% reduction" was an error — the 66% figure appeared to have been derived from a different calculation in the thesis and was imported incorrectly. It has been removed. The revised Discussion presents the Spearman correlation correctly and notes the binary nature of the measurement. | Revised Discussion: "The observation checklist recorded, each month, whether health workers had made any documented effort to identify and link defaulters — a binary (yes/no) item. Facilities where such efforts were documented consistently were associated with lower dropout rates (Spearman r = 0.354, p = 0.021). We caution against interpreting this as a measure of effort intensity; the checklist could not capture how much time or how many contacts were involved, only whether any effort occurred." The "66% reduction" claim has been removed. |

**Comment 31** — Discussion – Organisation

| **Editor Comment** | **Authors’ Response** | **Manuscript Change** |
| --- | --- | --- |
| In general, the discussion could be a bit better organized. It seems repetitive. | We agree that the original Discussion was repetitive and insufficiently structured. We have reorganised it into three clearly labelled sub-sections: (1) Monitoring charts and the value of routine data use, (2) Boma Health Workers and the community interface, and (3) Defaulter tracing systems and vaccine stockouts. Each sub-section presents the relevant findings, contextualises them against the literature, and draws a targeted inference. Repetitive passages that appeared in multiple sub-sections have been consolidated or removed. We have also adopted the editor's suggested framing ("our data are consistent with the findings of X, who showed...") to improve integration of the literature. | Revised Discussion: Three sub-sections with clear headings. Repetition removed. The framing "our data are consistent with [author], who..." is used to integrate literature throughout. |

**Section 6: Conclusion**

**Comment 32** — Conclusion – Vaccine Supply

| **Editor Comment** | **Authors’ Response** | **Manuscript Change** |
| --- | --- | --- |
| How does lack of vaccine supply fit into this? Are vaccines ordered based on the records? Are people whose children missed vaccination because of short supply then recalled when the vaccine comes in? | These are excellent questions that illuminate an important structural gap in the original paper. Ninety-three percent of respondents identified vaccine stockouts as a recognized cause of increased defaulters, yet the original manuscript did not explore the mechanisms by which stockouts translate into dropout or how monitoring data could be used to prevent them. We have addressed this in both the Discussion and Conclusions. Specifically, we note that HFMP population estimates and monthly coverage data (already collected by most facilities) could directly inform vaccine ordering, making stockouts a preventable event. Regarding recall after stockout resolution: our study did not directly measure this, and we acknowledge that as a gap. We note, however, that the defaulter tracing system — if used proactively — should capture children missed due to stockout in the same way as children who missed due to other reasons, enabling recall visits once vaccine supply is restored. | Revised Discussion (sub-section on defaulter tracing and vaccine supply): "Vaccine stockouts represent a structural threat that monitoring data alone cannot resolve...An important next step is to use HFMP population estimates and monthly coverage data to drive vaccine quantity ordering, so that stockouts become a predictable and preventable event."  Revised Conclusions: "...monitoring data — from immunization registers, HFMP population estimates, and monthly coverage summaries — should be directly linked to vaccine ordering processes to mitigate the stockout-driven dropout that 93% of respondents identified as a recognized problem." |

**Comment 33** — Conclusion – Definition of Monitoring

| **Editor Comment** | **Authors’ Response** | **Manuscript Change** |
| --- | --- | --- |
| "Monitoring charts and defaulter tracing system were less commonly used. Gaps of no monitoring chart in other health facilities were attributed to poor performance in monitoring of immunization services." A general problem in understanding this is that monitoring seems to mean use of the charts sometimes, but it really doesn't. So what IS monitoring and who does it? This could be better explained in the introduction/methods. | This is an important conceptual clarification. In the original manuscript, "monitoring" was used inconsistently to refer variously to: (a) the act of using the monitoring chart specifically; (b) the broader set of activities involved in tracking immunization coverage and defaulters; and (c) the immunization program as a whole. This ambiguity would confuse readers.  We have addressed this in the Introduction by providing an explicit working definition of "monitoring mechanisms" as used in this study: the systematic tools and processes — both facility-level (monitoring charts, immunization registers, defaulter tracing systems, HFMP documents) and community-level (BHW referral and tracing activities) — used to track vaccination coverage, identify defaulters, and prompt corrective action. We have also clarified who performs each type of monitoring: facility-based monitoring tools are used by vaccinators and health workers; community-level monitoring is carried out by BHWs who operate in the catchment area. This framing is maintained consistently throughout the revised manuscript. | Revised Introduction: An explicit definition is now provided: "For the purposes of this study, monitoring mechanisms refers to the systematic tools and processes — both facility-level and community-level — used to track immunization coverage, identify defaulters, and prompt corrective action."  Revised Methods: The two categories of monitoring are distinguished: facility-based tools (monitoring chart, registers, defaulter tracing system, HFMP) and community-based mechanisms (BHW referral and tracing).  Revised Conclusions: The ambiguous use of "monitoring" in the original conclusion has been replaced with specific references to the monitoring charts and defaulter tracing systems. |

**Summary of all revisions made**

| **#** | **Editor comment (abbreviated)** | **Section** | **Action taken** |
| --- | --- | --- | --- |
| 1 | Time point missing for "coverage reduced to 84%" | Abstract – Introduction | Years added: 2019→86%, 2020→84%, 2021→82% |
| 2 | Missing grammatical subject in abstract sentence | Abstract – Introduction | Incomplete sentence rewritten with explicit subject and temporal data |
| 3 | Abbreviation "BHWs" used before definition | Abstract – Methods/Results | "Boma Health Workers (BHWs)" spelled out on first use in abstract |
| 4 | Boma Health Worker undefined; study duration not stated | Abstract – Methods/Results | BHW defined inline; "1 October–31 December 2023" added |
| 5 | Internal vs. external monitoring not distinguished in conclusions | Abstract – Conclusions | Conclusions rewritten: facility-based tools vs. community-based BHW mechanisms |
| 6 | No time frame on global vaccine uptake reduction statement | Introduction | Years 2019/2020/2021 with corresponding coverage figures added |
| 7 | Repetitive sentence about monitoring mechanisms | Introduction | Sentence restructured; "monitoring mechanisms" appears once |
| 8 | "Nevertheless" used inappropriately | Introduction | Word removed; paragraph restructured for logical flow |
| 9 | "on a timely fashion," "provide," "reflect" — grammar errors | Introduction | Corrected to "in a timely fashion," "provides," "reflecting" |
| 10 | "toasted coin" — spell-checker error | Methods | Corrected to "coin toss" throughout |
| 11 | Erroneous comma between subject and relative clause | Methods | Comma removed |
| 12 | "Attributed to" implies reverse causality for BHW/dropout | Methods | "Attributed to" → "associated with" throughout; causal limitation noted in Discussion |
| 13 | IC language/translation protocol unspecified; direction incorrect | Methods | Corrected to "obtained from"; verbal translation protocol and ethics approval described |
| 14 | Frequency before parenthesis formatting error throughout | Results | All n(%) pairs reformatted to "n (%)" convention |
| 15 | "Respondent" ambiguous — who is responding? | Results | Clarified as "one vaccinator per facility" at first use |
| 16 | 100% male secondary education not explained; "respondent" singular | Results | Explained: all 11 males = secondary; all 3 females = primary. "Respondents" (plural) used |
| 17 | "tract" should be "track" | Results | Corrected throughout |
| 18 | "realize their defaulters" should be "identify" | Results | "Realize" → "identify" throughout |
| 19 | Were defaulter identification methods mutually exclusive? | Results | Clarified as non-mutually-exclusive; explanatory note added |
| 20 | Credibility of observation data; Hawthorne effect risk | Results + Limitations | Checklist described as record-based, not behavioral; Hawthorne effect acknowledged in Limitations |
| 21 | Causal language used for association finding | Results + Discussion | "Reducing" → "associated with"; causal limitation stated explicitly |
| 22 | "document been updated" — grammatically incorrect | Results | Corrected to "that had been recently updated" |
| 23 | Normative opinion in Results section | Results → Discussion | Sentence removed from Results; refined interpretive version added to Discussion |
| 24 | BHW paragraph needs a table | Results | Table 3 added: BHW status × dropout rate category; cited in text |
| 25 | Interpretive sentences about BHW mechanisms in Results | Results → Discussion | Removed from Results; incorporated into Discussion on BHW community interface |
| 26 | "50% effort" ambiguous: 50% of centres or 50% of effort? | Results | Clarified: 50% of facility-month observations showed documented effort; phrase "50% effort" removed |
| 27 | Defaulter tracing data duplicated; suggest a table | Results | Duplication removed; Table 4 added with all defaulter tracing data consolidated |
| 28 | Continuous phrasing for binary (have/have not) monitoring chart | Discussion | Rewritten: "Facilities that had a monitoring chart...were less likely to record high dropout" |
| 29 | "The study finding" — should be "findings" (plural) | Discussion | "Findings" (plural) used throughout |
| 30 | "66% reduction" overinterprets unmeasured effort variable | Discussion | Claim removed; Spearman r = 0.354 presented with explanation that "effort" is binary (yes/no) |
| 31 | Discussion disorganised and repetitive | Discussion | Restructured into 3 sub-sections; repetition removed; "our data are consistent with" framing adopted |
| 32 | How does vaccine supply relate to monitoring and dropout? | Conclusion + Discussion | New sub-section added on stockouts; monitoring data linked to ordering; recall gap acknowledged |
| 33 | Definition of "monitoring" is inconsistent; who does it? | Intro + Methods + Conclusion | Explicit definition added in Introduction; two categories (facility-level, community-level) defined and maintained throughout |

We believe the revised manuscript is substantially strengthened by these changes. We are grateful to the Academic Editor for the rigorous and detailed review, and we are happy to provide any further clarification or additional revisions if requested.

Respectfully submitted,

Daniel Opinile Mark Igga, MPH

On behalf of: Nzomo Mwita, PhD; Kennedy Muni, PhD

Corresponding author: Danielmarkigga@gmail.com | +211 921 825 525

Manuscript number: PONE-D-25-26100
