# Supplementary material for: Effectiveness of monitoring mechanisms in reducing immunization dropouts among infants in health facilities of Maridi County, South Sudan
Source: PLoS One. 2026 Sep 3;21(9):e0356268. doi: 10.1371/journal.pone.0356268 (PMC13541179; doi:10.1371/journal.pone.0356268)
Supplement: Supplementary file 29 [file pone.0356268.s033.docx]

**Response to Academic Editor Comments**

**Manuscript:** *Effectiveness of monitoring mechanisms in reducing immunization dropouts among infants in health facilities of Maridi County, South Sudan*

**Manuscript number:** PONE-D-25-26100	**|	Journal:** PLOS ONE

**Authors:** Daniel Opinile Mark Igga, Nzomo Mwita, Kennedy Muni

Dear Editor,

We thank the Academic Editor for a careful and constructive review. The comments identified real weaknesses in clarity, precision, and organization, and we have revised the manuscript thoroughly in response to every point.

Three points about this revision are worth flagging at the outset. First, we have converted the manuscript to the first person. The editor’s question about who obtained and interpreted the informed consent could not be answered cleanly while the manuscript referred to the investigator impersonally as “the researcher,” and that usage was in any case inconsistent with the occasional first-person phrasing elsewhere in the paper. The manuscript now uses “I” for work carried out by the first author alone (data collection, consent, observation) and “we” for judgements made by all three authors (interpretation, recommendations). This is addressed as Comment 13b below.

Second, every quotation in the third column of the tables below has been checked against the revised manuscript and reproduces its wording exactly.

Third, a version of the manuscript with all changes marked using Word’s track-changes feature accompanies this letter, redlined against the originally submitted version of PONE-D-25-26100, so that each change described below can be located directly in the text.

Yours sincerely,

Daniel Opinile Mark Igga, on behalf of all authors

**Section 1: Abstract**

**Comment 1** | Abstract: Introduction

| **Editor comment** | **Authors’ response** | **Change in manuscript (verbatim)** |
| --- | --- | --- |
| "coverage reduced to 84%". At what time point? | The editor is correct that the time point was missing, which left the figure unverifiable. Global third-dose pentavalent coverage was 86% in 2019, 84% in 2020, and 82% in 2021. All three years are now stated. | *"Global third-dose pentavalent coverage stood at 86% in 2019, declined to 84% in 2020, and fell further to 82% in 2021, consistently falling short of the 85% minimum target."* |

**Comment 2** | Abstract: Introduction

| **Editor comment** | **Authors’ response** | **Change in manuscript (verbatim)** |
| --- | --- | --- |
| "Despite the efforts employed to improve the immunization coverage, SUBJECT NEEDED HERE remained below 85%." | Agreed: the sentence had no grammatical subject. Rather than patch it, we removed the fragment and folded its substance into the preceding sentence, which now carries both the trend data and the comparison to target. | *The fragment has been deleted. The surviving sentence ends: "...consistently falling short of the 85% minimum target."* |

**Comment 3** | Abstract: Methods/Results

| **Editor comment** | **Authors’ response** | **Change in manuscript (verbatim)** |
| --- | --- | --- |
| "BHWs" — make sure the abbreviations are written out first time they are used. | Agreed. "BHW" appeared in the abstract without prior definition. It is now spelled out on first use, with the abbreviation following in parentheses. | *"Boma Health Workers (BHWs), community-level health personnel who mobilize, trace, and refer immunization defaulters, were attached to 64% of facilities."* |

**Comment 4** | Abstract: Methods/Results

| **Editor comment** | **Authors’ response** | **Change in manuscript (verbatim)** |
| --- | --- | --- |
| Explain in abstract (briefly) what a Boma Health Worker is, also state how long the study lasted (i.e. over what period of time did changes occur?) | Both points are well taken. The BHW is a cadre specific to South Sudan, so readers elsewhere have no frame of reference for the term; and without a time frame the reader cannot judge what the observed reduction means. Both are now given. | *Duration: "I conducted a prospective cohort study from 1 October to 31 December 2023..."*  *Definition: as quoted under Comment 3.*  *Trend: "Over the three months of observation, the proportion of facilities recording a dropout rate above 10% fell by 9.6 percentage points."* |

**Comment 5** | Abstract: Conclusions

| **Editor comment** | **Authors’ response** | **Change in manuscript (verbatim)** |
| --- | --- | --- |
| Can you distinguish between the monitoring tools used by internal health workers and the external monitoring and the effect of BHWs? | This distinction was conflated in the original. Monitoring charts, registers, and defaulter tracing cards are operated by health workers inside the facility; BHWs operate in the community. The abstract conclusions now separate the two and state that they are complementary rather than interchangeable. | *"Facility-based monitoring tools used by health workers (monitoring charts, immunization registers, and defaulter tracing systems) and community-based monitoring carried out by BHWs were each independently associated with lower dropout rates, and their combination appears to have a complementary effect. Neither mechanism was sufficient alone."* |

**Section 2: Introduction**

**Comment 6** | Introduction

| **Editor comment** | **Authors’ response** | **Change in manuscript (verbatim)** |
| --- | --- | --- |
| "The number of infants vaccinated globally reduced, specifically, uptake of 3rd dose of pentavalent." Statements like this need to have a time frame. The next sentence is not clear. | Agreed on both counts: no year was given, and the sentence did not complete a grammatical thought. The passage has been rewritten with explicit years, and the unclear follow-on sentence about the third global goal has been deleted rather than patched. | *"Third-dose pentavalent coverage, a widely used proxy for immunization system performance, stood at 86% globally in 2019 but slipped to 84% in 2020 and 82% in 2021, consistently falling short of the 85% floor target set by the Immunization Agenda 2030 [3]."* |

**Comment 7** | Introduction

| **Editor comment** | **Authors’ response** | **Change in manuscript (verbatim)** |
| --- | --- | --- |
| "The study intends to determine the use of immunization monitoring mechanism as a strategy to improve the county's performance..." This sentence is repetitive about using monitoring mechanisms. | Agreed: the phrase appeared twice in one sentence. The county coverage figures now stand alone as factual context, and the study aim is stated separately at the end of the Introduction. | *"According to 2022 programme data, third-dose pentavalent coverage in the county was 82% and measles vaccine coverage was 55%, both below the national 85% target."* |

**Comment 8** | Introduction

| **Editor comment** | **Authors’ response** | **Change in manuscript (verbatim)** |
| --- | --- | --- |
| Why "Nevertheless"? seems like it's all saying the same thing. | The editor is right: the word signalled a contrast that was not there. It has been deleted along with the sentence it introduced, which restated the preceding point. | *"Nevertheless" and the sentence following it have been deleted. The paragraph now ends: "...thereby increasing herd immunity and reducing the burden of vaccine-preventable disease."* |

**Comment 9** | Introduction

| **Editor comment** | **Authors’ response** | **Change in manuscript (verbatim)** |
| --- | --- | --- |
| "monthly goals on a timely fashion, provide basis for ...." should be "in" a timely fashion and "provideS" and "reflecting" | All three corrected. We note that the surrounding sentence was also rebuilt, so the corrections appear within rephrased text rather than as isolated word substitutions. | *"...enables immunization programme managers to set and evaluate monthly goals in a timely fashion, provides a basis for comparing performance across different time periods, monitors each vaccine and dose separately, and reflects the situation accurately [5]."* |

**Section 3: Materials and Methods**

**Comment 10** | Methods

| **Editor comment** | **Authors’ response** | **Change in manuscript (verbatim)** |
| --- | --- | --- |
| "vaccinator was selected based on toasted coin" should be based on a tossed coin or based on a coin toss (spell check helping you too much here!) | A spell-checker substitution we failed to catch. Corrected in both places it occurred. | *"A coin was tossed to select a vaccinator from each eligible health facility..." and, in the inclusion criteria, "...selected by the coin toss described above."* |

**Comment 11** | Methods

| **Editor comment** | **Authors’ response** | **Change in manuscript (verbatim)** |
| --- | --- | --- |
| "had a written informed consent, that" (no comma should be here between subject and verb) | Correct: the comma separated the noun from its relative clause. Removed. | *"The semi-structured interview guide had a written informed consent that explained the purpose of the research..."* |

**Comment 12** | Methods

| **Editor comment** | **Authors’ response** | **Change in manuscript (verbatim)** |
| --- | --- | --- |
| "This examined whether the presence of BHWs in health facilities was either attributed to having low dropout rates..." Perhaps rather than "attributed to", you meant to say "associated with". The sentence now seems to say that the dropout rate caused the BHW to be in the health facility. | The editor has identified a genuine reversal of the intended direction. We were testing whether BHW presence relates to dropout rate, not whether dropout rate determined BHW deployment. "Attributed to" has been replaced with "associated with" here and in the Conclusions, and we have added an explicit statement that the design supports association only. | *"This examined whether the presence of BHWs in health facilities was associated with low dropout rates (less than 10%) or high dropout rates (more than 10%)."*  *Added: "Because facilities were not randomized to condition, all results reported below are associations and should not be read as establishing causation."* |

**Comment 13a** | Methods: direction of consent and translation protocol

| **Editor comment** | **Authors’ response** | **Change in manuscript (verbatim)** |
| --- | --- | --- |
| "The researcher provided written informed consent to the participants." It seems that the researcher OBTAINED written informed consent FROM the participants. What was the original language of the IC? Please state whether the IC translation was written (and verified in some way and approved by ethics committee) or by a script that was oral (approved?), or whether it was done each time verbally by the person obtaining informed consent. | The direction was indeed reversed, and we apologise for the error. On the translation protocol, the editor offers three alternatives and we confirm it was the third: the consent form existed only in English, no written translations were produced and no fixed oral script was used, and the interpretation was delivered verbally, in person, on each separate occasion, by the same individual who obtained the consent, namely the first author. That protocol was described in the study protocol and approved by both ethics bodies. All four elements are now stated explicitly. | *"The consent form was prepared in English. Because participants varied in the language they were most comfortable with, I verbally interpreted the consent form into each participant's preferred language (Juba Arabic, Muru, Azande, Baka, or Avokaya) immediately before they signed. No written translations were produced; the interpretation was delivered by me, in person, on each occasion, and I obtained written informed consent from every participant myself. This verbal interpretation protocol was described in the study protocol and approved by the Amref International University Ethics and Scientific Review Committee and the South Sudan Ministry of Health Research Ethics Review Board."* |

**Comment 13b** | Whole manuscript: first-person voice (new in this revision)

| **Editor comment** | **Authors’ response** | **Change in manuscript (verbatim)** |
| --- | --- | --- |
| Arising from the comment above: "...or whether it was done each time verbally by the person obtaining informed consent." | Answering the editor's question precisely required naming who performed the act, which the manuscript's impersonal "the researcher" made awkward. On reviewing the paper we also found the voice was internally inconsistent: "the researcher" appeared four times while "our data" appeared in the Discussion. We have therefore converted the manuscript to the first person throughout, following PLOS ONE style, which permits it. We use "I" for work the first author carried out alone (obtaining consent, interpreting it, administering interviews, completing the observation checklist, and holding the data) and "we" for judgements shared by all three authors, such as interpretation of findings, caveats, and recommendations. No third-person self-reference remains. | *Representative changes: "I conducted a prospective cohort study from 1 October to 31 December 2023..."; "I obtained written informed consent from every participant before data collection began..."; "All the research data were kept safely by me for future reference, identified by participant number rather than name."; "The observation checklist was completed by me during facility visits..."; "We therefore make no claim about the amount of effort involved..."; "We recommend that health workers use monitoring tools consistently..."* |

**Section 4: Results**

**Comment 14** | Results: number formatting

| **Editor comment** | **Authors’ response** | **Change in manuscript (verbatim)** |
| --- | --- | --- |
| "Out of the 14 health facilities, (12, 86%) were supported..." The 12 should be before parentheses. Same for the 2 later on in the sentence, etc etc. | Agreed; "n (%)" is the correct convention. Every frequency-percentage pair in the Results has been reformatted. | *"...12 (86%) were supported by non-governmental organizations while 2 (14%) were supported by the Government." The same correction is applied to all subsequent pairs.* |

**Comment 15** | Results: meaning of "respondent"

| **Editor comment** | **Authors’ response** | **Change in manuscript (verbatim)** |
| --- | --- | --- |
| "Majority of the male respondents (11, 79%)...." Be clear that you mean by respondent the person responding on behalf of the health facility. | Agreed: the term was ambiguous. A definition now appears at first use in the Results, before any respondent figures are reported. | *"Throughout this section, 'respondent' denotes the single vaccinator selected to respond on behalf of each health facility; there were therefore 14 respondents, one per facility."* |

**Comment 16** | Results: education by sex

| **Editor comment** | **Authors’ response** | **Change in manuscript (verbatim)** |
| --- | --- | --- |
| The % of males who had secondary education seems to be 100%. The % of all respondents who had secondary education is 79%. Does this mean there were just 3 women responding? Make that respondentS. | The editor's inference is correct: there were 3 female respondents, and all had primary education only, while all 11 male respondents had secondary education. The original wording obscured this. The plural is now used, the breakdown is stated explicitly, and Table 1 has been added so the counts can be read directly. | *"Eleven respondents (79%) were male and 3 (21%) were female... Disaggregated by sex, all 11 male respondents had attained secondary-level education, while all 3 female respondents had attained primary-level education only." Table 1 (Demographic characteristics of respondents, N = 14) is new.* |

**Comment 17** | Results: typographical

| **Editor comment** | **Authors’ response** | **Change in manuscript (verbatim)** |
| --- | --- | --- |
| "Use of Immunization monitoring chart to tract immunization performance." Should be TRACK | Corrected. The heading has also been put into sentence case per PLOS ONE style. | *Heading now reads: "Use of immunization monitoring charts to track immunization performance"* |

**Comment 18** | Results: word choice

| **Editor comment** | **Authors’ response** | **Change in manuscript (verbatim)** |
| --- | --- | --- |
| "(1, 7%) used monitoring chart to realize their defaulters." Should be "recognize" or "identify", not realize. | Agreed; "identify" is the standard term and is now used. | *"...and 1 (7%) used the monitoring chart to identify defaulters."* |

**Comment 19** | Results: overlapping categories

| **Editor comment** | **Authors’ response** | **Change in manuscript (verbatim)** |
| --- | --- | --- |
| Are these methods of identifying defaulters overlapping (more than one used) or exclusive categories (either/or)? | They overlap: several facilities used more than one tool, so the percentages do not sum to 100%. The original text implied exclusivity by omission. This is now stated before the figures are given. | *"Respondents were not restricted to a single answer, and several facilities used more than one tool; the categories below therefore overlap and do not sum to 100%."* |

**Comment 20** | Results/Methods: credibility of observation data

| **Editor comment** | **Authors’ response** | **Change in manuscript (verbatim)** |
| --- | --- | --- |
| What does "the observation data" mean? For the discussion, how credible are these data? Were people trying extra hard when observers were there? Was there another way to validate the data? | A fair challenge. "Observation data" meant data recorded on the monthly observation checklist, as distinct from self-report at interview. On credibility: the checklist items were transcribed from the facility's own monthly records rather than from watching staff work, so they reflect performance across the whole month rather than conduct during the visit, which limits, without eliminating, the effect the editor describes. We did not carry out an independent re-abstraction of those records, and we now say so rather than imply a validation that did not occur. The residual risk is also carried into the Limitations. | *Methods: "The checklist items were transcribed from records the facility maintains for its own reporting (the monthly summary report, the immunization registers, and the monitoring chart) rather than from real-time observation of health worker behaviour. Because those records document performance accumulated across the whole month, rather than conduct at the moment of the visit, this reduces (though it does not eliminate) the risk that facilities appeared to perform better simply because they knew I was present. No independent re-abstraction of the records was carried out, and this is noted as a limitation."* |

**Comment 21** | Results: causal language

| **Editor comment** | **Authors’ response** | **Change in manuscript (verbatim)** |
| --- | --- | --- |
| "... was statistically significant (Mean difference: 1.78, 95% CI: 1.22 - 2.35) in reducing defaulters in the health facilities." Use of monitoring tools was ASSOCIATED with a lower rate of defaulters. This does not prove it was the cause, although that may well be true (to talk about in the Discussion). | Accepted without reservation. Significance establishes association, not cause, in an unrandomised design. The phrase "in reducing defaulters" has been deleted here, similar phrasing corrected throughout, and the limitation stated in both Methods and Discussion. | *"Use of monitoring tools to identify immunization defaulters was significantly associated with a lower rate of defaulters (Mean difference: 1.78, 95% CI: 1.22 - 2.35)."*  *Limitations: "...because facilities were not randomized to condition, the associations reported here cannot establish causation."* |

**Comment 22** | Results: grammar

| **Editor comment** | **Authors’ response** | **Change in manuscript (verbatim)** |
| --- | --- | --- |
| "health facility microplanning document been updated," should say "being updated". Or "that had been updated". | Corrected using the second of the editor's suggestions, and the following clause adjusted to agree. | *"The use of monitoring tools was associated with the health facility microplanning document having been updated, with a 54% coefficient of association and p-value 0.04, indicating that the more the microplanning documents were updated the more likely monitoring tools were used..."* |

**Comment 23** | Results → Discussion: opinion misplaced

| **Editor comment** | **Authors’ response** | **Change in manuscript (verbatim)** |
| --- | --- | --- |
| "As much as monitoring chart is a performance tool, it should be used to display the performance and gauge the success of immunization rather than identifying the immunization defaulters." This sentence expresses a general opinion and should be in the Discussion section, not results. | Agreed: this is an interpretation, not a finding. It has been deleted from the Results and a fuller version placed in the Discussion, where we also note that most respondents were in fact already using the tools this way. | *Deleted from Results. Discussion now reads: "It is worth noting here that the monitoring chart is a performance tool: it is designed to display aggregate coverage and gauge the success of the immunization programme, rather than to identify individual defaulters, for which the register and the defaulter tracing card are the appropriate instruments. Most respondents used the tools in exactly this way, which is a positive indicator of monitoring tool literacy..."* |

**Comment 24** | Results: BHW paragraph needs a table

| **Editor comment** | **Authors’ response** | **Change in manuscript (verbatim)** |
| --- | --- | --- |
| Presence of BHWs in the health facilities is a key factor.... This paragraph would be well illustrated by a table to be referred to in the text. | Agreed. Table 3 has been added, cross-tabulating BHW attachment against dropout rate category across the 42 facility-month observations, and is cited in the text at that point. The narrative has been shortened accordingly so the table carries the detail. | *New Table 3: "BHW attachment status in relation to dropout rate category across 42 facility-month observations", cited as "Table 3 presents the relationship between BHW attachment and dropout rate category."* |

**Comment 25** | Results → Discussion: mechanism and motivation

| **Editor comment** | **Authors’ response** | **Change in manuscript (verbatim)** |
| --- | --- | --- |
| "Presence of BHWs attached to the health facilities should facilitate referrals of the defaulters... The health workers felt motivated to support the tracing the defaulters as BHWs supported them in ensuring that....." This belongs in the discussion. | Agreed. The first sentence proposes a mechanism and the second reports a subjective impression; neither is a result. Both have been removed from the Results and the substance relocated to the Discussion. | *Deleted from Results. Discussion now reads: "The mechanism we observed is a two-sided one: health workers identify defaulters and hand over a list, and BHWs locate those children in the community and refer them back. Health workers reported feeling supported in tracing defaulters when BHWs were available to help find the infants."* |

**Comment 26** | Results: ambiguous "50% effort"

| **Editor comment** | **Authors’ response** | **Change in manuscript (verbatim)** |
| --- | --- | --- |
| "had fifty percent (50%) effort to identify the defaulters and trace them." Does this mean 50% of the centers attempted to identify defaulters? Or that all of them tried half as hard? | Neither reading was excluded by the original wording. The intended meaning is the first, at the level of facility-months: among facilities above 10% dropout, effort was recorded in half of the months observed and absent in the other half. The phrase "50% effort" has been removed and the sentence rewritten, with a note that the item was binary. | *"...while facilities with more than 10% dropout rates had documented effort to identify and trace defaulters recorded in only 50% of their facility-month observations; that is, in half of the months observed at those facilities, no such effort was recorded at all. The checklist captured this as a binary yes/no item and did not measure how much effort was expended."* |

**Comment 27** | Results: duplication

| **Editor comment** | **Authors’ response** | **Change in manuscript (verbatim)** |
| --- | --- | --- |
| "Ninety three percent (13, 93%) of the respondents confirmed that their facilities have defaulter tracing system,..." These data were also mentioned earlier, but it is much clearer in this paragraph. Suggest eliminating the duplication and showing this in a table, referred to in text. | Agreed. The earlier partial statement has been deleted and all defaulter tracing figures consolidated into a single paragraph supported by Table 4. | *New Table 4: "Defaulter tracing system characteristics and related practices (N = 14 facilities)", covering tracing system presence, card completion, tickler file knowledge, stockout recognition, and HFMP update status. Text now closes: "These findings are summarized in Table 4."* |

**Section 5: Discussion**

**Comment 28** | Discussion: association and binary phrasing

| **Editor comment** | **Authors’ response** | **Change in manuscript (verbatim)** |
| --- | --- | --- |
| "the more health facilities have monitoring charts, the lesser they would have dropouts". Agree with the sentiment here, but it is an association, not proven (suggests that) and also not written clearly. They do or not, yes or no, correct? But this is written as if each facility can have more or less of a chart. | Both objections are correct. A facility either has a chart or does not, so the comparative construction was inappropriate; and the claim was stated as established rather than as an association. The sentence has been rewritten to treat chart presence as binary, to distinguish presence from updating, and to state the inferential limit. | *"Facilities that had a monitoring chart, and particularly those that kept it updated, were less likely to record a dropout rate above 10% (r = -0.725, p = 0.003). Because facilities were not randomized, this is an association rather than a demonstrated cause."* |

**Comment 29** | Discussion: grammar

| **Editor comment** | **Authors’ response** | **Change in manuscript (verbatim)** |
| --- | --- | --- |
| "The study finding agreed...." The study has findingS | Corrected. We have also adopted the editor's suggested construction for citing prior work (see Comment 31). | *"The findings are also consistent with those of Shikuku et al. [7], who showed that..."* |

**Comment 30** | Discussion: overinterpretation of "effort"

| **Editor comment** | **Authors’ response** | **Change in manuscript (verbatim)** |
| --- | --- | --- |
| "A relationship existed between health workers' effort to identify defaulters... it reduces the dropout rate by 66%." How did you identify the amount of effort each health worker put into the identification of defaulters? I didn't see quantitative data, which would be hard or impossible to get. So this statement seems to overinterpret your results. It is probably true, but did you measure the amount of effort? | The editor is right, and we withdraw the claim. We did not measure the amount of effort. The checklist asked only whether any effort to identify and link defaulters was documented in a given month, a yes/no item. The 66% figure was carried across from a different calculation in the underlying thesis and does not describe this variable at all; it has been removed. The passage now reports the Spearman coefficient, states plainly what the item did and did not capture, and records the withdrawal so the change is visible to readers of both versions. | *"Facilities where health workers made documented efforts to identify defaulters were associated with lower dropout rates (Spearman r = 0.354, p = 0.021). This measure requires an important caveat: the observation checklist recorded only whether any effort to identify and link defaulters occurred in a given month, as a binary yes/no item. It did not quantify how much effort was expended, how many households were visited, or how many contacts were attempted, and we did not collect data that would support such a measure. We therefore make no claim about the amount of effort involved, and the earlier framing of this result as a 66% reduction in dropout has been withdrawn as an overinterpretation."* |

**Comment 31** | Discussion: organisation

| **Editor comment** | **Authors’ response** | **Change in manuscript (verbatim)** |
| --- | --- | --- |
| In general, the discussion could be a bit better organized. It seems repetitive.  A good way to express some of these thoughts might be to say "our data are consistent with the findings of so-and-so, who showed....". | Agreed. The Discussion has been reorganised under four headed sub-sections (monitoring charts and routine data use; BHWs and the community interface; microplanning, defaulter tracing and vaccine supply; and limitations), with repeated material consolidated. We have adopted the editor's suggested formulation throughout for integrating prior work. | *Sub-headings added. The suggested construction is used at each citation, e.g. "Our data are consistent with the findings of Chantler and colleagues [6], who showed that..." and "Our data are consistent with the findings of Samuel [9], who showed that..."* |

**Section 6: Conclusion**

**Comment 32** | Conclusion: vaccine supply

| **Editor comment** | **Authors’ response** | **Change in manuscript (verbatim)** |
| --- | --- | --- |
| How does lack of vaccine supply fit into this? Are vaccines ordered based on the records? Are people whose children missed vaccination because of short supply then recalled when the vaccine comes in? | These questions exposed a genuine gap. Taking them in turn: vaccine quantities are forecast from the catchment population held in the HFMP document and from consumption recorded in the registers, which are the same records that feed the monitoring chart, so weak record-keeping degrades ordering and defaulter identification simultaneously, which may explain why the two moved together in our data. On recall: a child turned away during a stockout should be carded and recalled like any other defaulter, but we did not measure whether this happened, and we now say so plainly rather than assert it. A new paragraph in the Discussion sets this out and the recommendation follows in the Conclusions. | *Discussion: "Vaccine quantities for a facility are forecast from the catchment population estimate held in the HFMP document and from monthly consumption recorded in the registers, which are the same records that feed the monitoring chart... A child turned away during a stockout becomes a defaulter like any other, and should in principle be entered on a defaulter tracing card and recalled once supply is restored. We did not measure whether such recall actually occurred, and this is a gap that future work should address."*  *Conclusions: "We further recommend that monitoring data, including HFMP population estimates, register consumption figures, and monthly coverage summaries, be linked directly to vaccine ordering, so that stockout-driven dropout becomes a predictable and preventable event..."* |

**Comment 33** | Whole manuscript: what "monitoring" means and who does it

| **Editor comment** | **Authors’ response** | **Change in manuscript (verbatim)** |
| --- | --- | --- |
| "Monitoring charts and defaulter tracing system were less commonly used. Gaps of no monitoring chart in other health facilities were attributed to poor performance in monitoring of immunization services." A general problem in understanding this is that monitoring seems to mean use of the charts sometimes, but it really doesn't. So what IS monitoring and who does it? This could be better explained in the introduction/methods. | This was the most substantive of the editor's comments and we are grateful for it. The manuscript had been using "monitoring" to mean three different things: chart use specifically, the wider set of coverage- and defaulter-tracking activities, and the immunization programme generally. We have added a definition paragraph to the Introduction that states what we mean by the term and, critically, distinguishes the two categories by who operates them. That distinction is then held consistently through the Methods, Results, Discussion, and Conclusions, and the vague sentence quoted by the editor has been replaced. | *Introduction: "For the purposes of this study, monitoring mechanisms refers to the systematic tools and processes used to track vaccination coverage, identify defaulters, and prompt corrective action. These fall into two categories, distinguished by who operates them. Facility-level monitoring is carried out by vaccinators and health workers inside the health facility, using the immunization monitoring chart, immunization registers, defaulter tracing cards and tickler files, and the health facility microplanning (HFMP) document. Community-level monitoring is carried out by BHWs in the facility catchment area, and consists of household mobilization, tracing of children identified as defaulters, and referral of those children back to the facility. This distinction is maintained throughout the paper."*  *Conclusions, replacing the quoted sentence: "Facility-level monitoring (the monitoring chart, the immunization register, and the defaulter tracing system, operated by vaccinators and health workers) and community-level monitoring, operated by BHWs, were each associated with lower immunization dropout rates over the three-month study period."* |

**Summary of revisions**

| **#** | **Editor comment** | **Section** | **Action** |
| --- | --- | --- | --- |
| 1 | Time point missing for "reduced to 84%" | Abstract | Years 2019 / 2020 / 2021 stated with figures |
| 2 | Sentence missing a subject | Abstract | Fragment deleted; substance merged into preceding sentence |
| 3 | "BHWs" undefined at first use | Abstract | Spelled out with abbreviation in parentheses |
| 4 | BHW not explained; duration not stated | Abstract | BHW defined inline; study period 1 Oct to 31 Dec 2023 added |
| 5 | Internal vs external monitoring not distinguished | Abstract | Conclusions rewritten to separate the two and note complementarity |
| 6 | No time frame for global reduction | Introduction | Years added; unclear follow-on sentence deleted |
| 7 | Repetitive "monitoring mechanisms" | Introduction | Sentence split; phrase now appears once |
| 8 | "Nevertheless" signals a false contrast | Introduction | Word and redundant sentence deleted |
| 9 | "on a timely fashion" / "provide" / "reflect" | Introduction | All three corrected within rebuilt sentence |
| 10 | "toasted coin" | Methods | Corrected to "tossed" / "coin toss" in both places |
| 11 | Comma between subject and verb | Methods | Comma removed |
| 12 | "attributed to" reverses causal direction | Methods | Changed to "associated with"; non-causal design stated |
| 13a | Consent direction; translation protocol unspecified | Methods | Direction corrected; verbal in-person protocol and ethics approval stated |
| 13b | Who obtained the consent (arising) | Whole manuscript | Converted to first person: "I" for fieldwork, "we" for judgements |
| 14 | "(12, 86%)" formatting | Results | All pairs reformatted to n (%) |
| 15 | "Respondent" ambiguous | Results | Defined at first use as the vaccinator representing the facility |
| 16 | 3 women? "respondentS" | Results | Breakdown stated explicitly; Table 1 added |
| 17 | "tract" | Results | Corrected to "track"; heading put in sentence case |
| 18 | "realize their defaulters" | Results | Corrected to "identify" |
| 19 | Overlapping or exclusive categories? | Results | Stated as overlapping; note that percentages exceed 100% |
| 20 | Credibility of observation data | Methods / Limitations | Provenance stated; no re-abstraction admitted; residual risk in Limitations |
| 21 | Causal language for an association | Results / Discussion | "in reducing defaulters" deleted; association language throughout |
| 22 | "document been updated" | Results | Corrected to "having been updated" |
| 23 | Opinion in Results | Results → Discussion | Sentence deleted; fuller version placed in Discussion |
| 24 | BHW paragraph needs a table | Results | Table 3 added and cited |
| 25 | Mechanism and motivation in Results | Results → Discussion | Both sentences deleted; substance relocated |
| 26 | "50% effort" ambiguous | Results | Rewritten as 50% of facility-months; binary item noted |
| 27 | Defaulter tracing data duplicated | Results | Duplication removed; Table 4 added |
| 28 | Comparative phrasing for a binary variable | Discussion | Rewritten as binary; association limit stated |
| 29 | "finding" should be plural | Discussion | Corrected |
| 30 | "66% reduction" overinterprets effort | Discussion | Claim withdrawn; binary nature of the item stated |
| 31 | Discussion repetitive and unstructured | Discussion | Four sub-headings; "our data are consistent with..." adopted |
| 32 | How does vaccine supply fit in? | Discussion / Conclusion | New paragraph on ordering and recall; unmeasured recall admitted |
| 33 | What IS monitoring and who does it? | Intro / Methods / Conclusion | Definition paragraph added; two categories held throughout |

We believe the manuscript is substantially stronger for these changes, and we are happy to provide any further clarification the editor may require.

Respectfully submitted,

Daniel Opinile Mark Igga, MPH, on behalf of Nzomo Mwita, PhD and Kennedy Muni, PhD

Danielmarkigga@gmail.com | +211 921 825 525 | PONE-D-25-26100
