# Supplementary material for: Effectiveness of monitoring mechanisms in reducing immunization dropouts among infants in health facilities of Maridi County, South Sudan
Source: PLoS One. 2026 Sep 3;21(9):e0356268. doi: 10.1371/journal.pone.0356268 (PMC13541179; doi:10.1371/journal.pone.0356268)
Supplement: Peer review history

# Peer review: Decision Letter 0

**DOI:** 10.1371/journal.pone.0356268.r001

**Authors:** Patricia Fast

18 Nov 2025

Dear Dr. Igga,

Thank you for submitting your manuscript to PLOS ONE. After careful consideration, we feel that it has merit but does not fully meet PLOS ONE’s publication criteria as it currently stands. Therefore, we invite you to submit a revised version of the manuscript that addresses the points raised during the review process.

==============================

The manuscript requires extensive rewriting in order to be fully evaluated.  Please look carefully at Reviewer !'s comments, in particular the suggestion to use the STROBE guidelines and checklists for the presentation of observational and qualitative studies.  The most important critique is that the methods are not clearly described, particularly how the analysis of the qualitative interviews was done.  Also, kindly present a much clearer definition of 'Monitoring': who does it and how do they measure the results?  It is not,required that the results be 'novel', only that the research be well described and the results support the conclusions.

The attached data are helpful, however some tables should be incorporated in the manuscript and even if they remain in the supplement, it is necessary to refer to them in the manuscript, so the reader can understand your process.  The first supplementary data are in fact tables, not figures.  Likewise, the qualitative data are not shown.

Please describe how you have carried out your analysis.  The tables are presented by topic, but what is not shown is the interrelationships between these data as they relate to the individual helath centers.  So some of the statements in the paper, about how effort relates to defaulter rate, is not supported by the data as it is provided.  Another small example is that you state the male reporteds had secondary education and the females did not, but that can't be discovered from the two separate tables you show.  A reader should be able to reproduce your statistical calculations by looking at your data, and that does not seem possible.

I have provided additional comments (see attachment).  Many of these are grammatical suggestions or suggested wording changes to increase clarity,

==============================

Please submit your revised manuscript by Jan 02 2026 11:59PM. If you will need more time than this to complete your revisions, please reply to this message or contact the journal office at plosone@plos.org. When you're ready to submit your revision, log on to https://www.editorialmanager.com/pone/ and select the 'Submissions Needing Revision' folder to locate your manuscript file.

- A rebuttal letter that responds to each point raised by the academic editor and reviewer(s). You should upload this letter as a separate file labeled 'Response to Reviewers'.
- A marked-up copy of your manuscript that highlights changes made to the original version. You should upload this as a separate file labeled 'Revised Manuscript with Track Changes'.
- An unmarked version of your revised paper without tracked changes. You should upload this as a separate file labeled 'Manuscript'.

If you would like to make changes to your financial disclosure, please include your updated statement in your cover letter. Guidelines for resubmitting your figure files are available below the reviewer comments at the end of this letter.

If applicable, we recommend that you deposit your laboratory protocols in protocols.io to enhance the reproducibility of your results. Protocols.io assigns your protocol its own identifier (DOI) so that it can be cited independently in the future. For instructions see: https://journals.plos.org/plosone/s/submission-guidelines#loc-laboratory-protocols. Additionally, PLOS ONE offers an option for publishing peer-reviewed Lab Protocol articles, which describe protocols hosted on protocols.io. Read more information on sharing protocols at https://plos.org/protocols?utm_medium=editorial-email&utm_source=authorletters&utm_campaign=protocols.

We look forward to receiving your revised manuscript.

Kind regards,

Patricia Evelyn Fast, MD, Ph.D.

Academic Editor

PLOS ONE

Journal requirements:

When submitting your revision, we need you to address these additional requirements.

1. Please ensure that your manuscript meets PLOS ONE's style requirements, including those for file naming. The PLOS ONE style templates can be found at

https://journals.plos.org/plosone/s/file?id=wjVg/PLOSOne_formatting_sample_main_body.pdf and

https://journals.plos.org/plosone/s/file?id=ba62/PLOSOne_formatting_sample_title_authors_affiliations.pdf

2. PLOS requires an ORCID iD for the corresponding author in Editorial Manager on papers submitted after December 6th, 2016. Please ensure that you have an ORCID iD and that it is validated in Editorial Manager. To do this, go to ‘Update my Information’ (in the upper left-hand corner of the main menu), and click on the Fetch/Validate link next to the ORCID field. This will take you to the ORCID site and allow you to create a new iD or authenticate a pre-existing iD in Editorial Manager.

3. If the reviewer comments include a recommendation to cite specific previously published works, please review and evaluate these publications to determine whether they are relevant and should be cited. There is no requirement to cite these works unless the editor has indicated otherwise.

Additional Editor Comments:

Thank you for submitting your work on this important topic. It requires extensive rewriting in order to be fully evaluated. Please look carefully at Reviewer !'s comments, in particular the suggestion to use the STROBE guidelines and checklists for the presentation of observational and qualitative studies. The most important critique is that the methods are not clearly described, particularly how the analysis of the qualitative interviews was done. Also, kindly present a much clearer definition of 'Monitoring': who does it and how do they measure the results? Please either incorporate the Revewer's comments or respond in a letter.

It is not, however, required that the results be 'novel', only that the research be well described and the results support the conclusions.

I have provided additional results (see attachment). Many of these are grammatical suggestions or suggested wording changes to increase clarity, and it is not requred to respond to these. However, there are a few more substantive comments or remarks that should be incorporated if possible in your revision.

The attached data are helpful, however some tables should be incorporated in the manuscript and even if they remain in the supplement, it is necessary to refer to them in the manuscript, so the reader can understand your process. The first supplementary data are in fact tables, not figures. Likewise, the qualitative data are not shown.

Please describe how you have carried out your analysis. The tables are presented by topic, but what is not shown is the interrelationships between these data as they relate to the individual helath centers. So some of the statements in the paper, about how effort relates to defaulter rate, is not supported by the data. Another small example is that you state the male reporteds had secondary education and the females did not, but that can't be discovered from the two separate tables you show. A reader should be able to reproduce your statistical calculations by looking at your data, and that does not seem possible.

[Note: HTML markup is below. Please do not edit.]

Reviewers' comments:

Reviewer's Responses to Questions

**Comments to the Author**

1. Is the manuscript technically sound, and do the data support the conclusions?

Reviewer #1: No

**********

2. Has the statistical analysis been performed appropriately and rigorously?

Reviewer #1: No

**********

3. Have the authors made all data underlying the findings in their manuscript fully available?

The PLOS Data policy

Reviewer #1: No

**********

4. Is the manuscript presented in an intelligible fashion and written in standard English?

Reviewer #1: Yes

**********

Reviewer #1: Several comments on the abstract:

Introduction – include more South Sudan-specific information.

Methods and conclusions – the study design cannot establish mechanisms of causality or assess effectiveness, which can only be done in randomized controlled trials. Who are the participants in the study? Vaccinators? How was the primary outcome defined? Stratified based on what? Further define what the exposed and unexposed groups of the cohort study are.

Results – no qualitative results were mentioned. Define BHWs. Unclear if dropout rates are measured per facility, per child, or per cohort.

Conclusion – The recommendation of scale-up is not supported by the data.

Introduction:

I recommend that the authors consult the STROBE checklist for cohort studies and include relevant items throughout their paper.

Authors should find more updated statistics about childhood immunization. The outdated 2019 statistics in the introduction should be replaced with more recent figures, as we know the COVID-19 pandemic reduced global childhood immunization coverage. Data from 2024 are available, so we would expect recent data to be incorporated in the introduction when referring to coverage and 0-dose children. For example, consider https://www.who.int/news-room/fact-sheets/detail/immunization-coverage that incorporates 2024 data. Additionally, authors cite an outdated version of IA2030 (the 2024 version is available). Moreover, 2024 estimates from South Sudan DTP1 coverage are available. Need references for immunization coverage rates.

The dropout rate is not defined. The dropout rate should have a clear operational definition that can be applied across settings.

Why do the authors switch from talking about immunization coverage to measles antigen performance without talking about measles coverage?

It’s unclear exactly what immunization monitoring mechanisms have worked or are being considered in the study. The specifics of what is being monitored, how, by whom, etc., need to be discussed. Also, what is the evidence of immunization monitoring for child vaccines? What has worked, and what are the gaps in evidence? Why have the authors focused on health facility microplanning and defaulter tracing systems? Authors should define what those mean.

Immunization dropout is defined as a child receiving at least one vaccine but not receiving subsequent vaccines. What about zero-dose vaccine children, who have never received immunizations? This is a common problem, which has gotten worse during and after the COVID-19 pandemic. How does this article address children who are unimmunized?

Authors cannot assess the effectiveness of any intervention with their study design. They are overstating their conclusions.

More details about the boma health initiative are warranted, such as who is involved, how it is implemented, and what is assessed.

Methods:

STROBE checklist 5 not clearly described (description of the setting, location, dates of exposure, dates of follow up and dates of data collection).

In addition, since this was also a qualitative study, I would like to see more details from the qualitative study reported based on the COREQ 32-item checklist of interviews and focus groups.

How were the 14 health facilities selected? Determining the sample size is not the same as selecting the health facilities.

Who are the participants?

STROBE checklist 7 and 8 have not been defined – we need to know all the outcomes, exposures, predictors, confounders, effective modifiers, and the sources of data and details of methods of measurement.

Show your sample size calculation. What assumptions were made?

Typo toasted

Since individuals from the same job often have different perspectives, I would have loved to see the authors interview all the vaccinators in the health facilities.

More details about the methodology of the qualitative study per the COREQ checklist. needs to be included. For example, items 9 through 23 of the checklist need to be addressed. In addition, how was the semi-structured interview guide developed?

What’s not clear is what the authors extracted and input into Excel and SPSS when this is largely a qualitative study.

The manuscript appears to conflate qualitative and quantitative analyses without clearly describing the methods used for each. It seems that this is a mixed-methods study, but that is not explicitly stated. The authors should clarify what quantitative analyses were conducted and specify what quantitative data were collected.

Author-state bivariable analysis was used to determine relationships, but that is not a statistical test. Was this logistical regression? Please expand on the statistical analysis for the quantitative part.

The authors report collecting data on immunization registers, health facilities, microplanning, and monitoring charts. However, the manuscript does not clearly describe how these variables were defined, operationalized, or measured. Given that these are central to the analysis, the authors should provide a detailed explanation of the data sources and collection methods for each variable to ensure clarity and reproducibility.

The authors should also specify the statistical measure used in these analyses (e.g., risk ratio or odds ratio), as this is not currently explained.

The manuscript does not provide sufficient detail on key data sources and tools. The authors should describe what the immunization registers contain and how information from them was used in the analysis. Similarly, the purpose and content of tickler files, defaulter tracing cards, and monitoring charts are not explained. Providing clear descriptions of these instruments is essential for readers to understand the data collection process and to assess the validity and replicability of the findings.

Results

The manuscript would benefit from the inclusion of tables or figures to summarize the data. For instance, demographic and facility characteristics could be presented in a descriptive table to enhance clarity. In addition, a table summarizing the quantitative regression analyses is expected.

The stated objective of the study is to ascertain the effectiveness of monitoring mechanisms in reducing immunization dropouts across health facilities. However, it is not clear how the presented analysis directly addresses or measures this objective. The association between the use of micro-monitoring tools and the updating of micro-planning documents, while potentially relevant, does not on its own demonstrate an effect on immunization dropout rates. The authors should clarify how this analysis contributes to assessing the effectiveness of monitoring mechanisms in reducing dropouts, and, if applicable, describe the analytical pathway linking these intermediate measures to the primary outcome.

The authors appear to include interpretive statements rather than presenting only the findings. For instance, explanations such as “accurate data capturing into the immunization register enhanced the completeness of the data tools” represent interpretation rather than results. Such explanatory or inferential statements would be more appropriately placed in the Discussion section, while the Results should focus on objectively reporting the observed data.

Based on the semi-structured interview data, the manuscript would benefit from a table summarizing the main themes accompanied by illustrative quotations to substantiate the findings. Similarly, a table summarizing the key results from the observational data is expected. The inclusion of these tables would enhance transparency, allow readers to assess the depth and credibility of the qualitative analysis, and improve the overall clarity of the results.

How did the authors address spillover in the locations between exposed and unexposed groups?

The manuscript should provide specific data on the actions taken by health facilities with desirable dropout rates to identify and trace defaulters. The current description of “effort” is vague and not operationally defined or measurable. The authors should clarify what specific activities or strategies were implemented and how these were assessed to allow for meaningful interpretation and comparison across facilities. The authors should define how they measured or categorized “effort.” For example: Low effort: No systematic review or tracing activities. Moderate effort: Passive record review without follow-up. High effort: Active tracing using multiple methods (register review, community outreach, phone calls).

Discussion

The study's novelty is not clearly established. The finding that monitoring immunization data can support tracking progress and reduce dropout rates is conceptually expected and has been well documented in prior literature. The authors should clarify what new insights their study provides beyond confirming that facilities that use monitoring tools perform better. For example, does the study offer a unique methodological approach, a deeper contextual understanding, or implementation insights that advance current knowledge? Clearly articulating the study’s added value would strengthen its contribution to the field.

The study needs to clarify its contribution beyond confirming that monitoring tools are associated with better immunization performance. The authors should explicitly articulate what is novel about their findings (e.g., a new implementation model, context-specific barriers, or unique operational lessons) and align their analytical approach with the stated objective of assessing effectiveness rather than mere association.

New findings are being added to the conclusion, including vaccine supply, which has not been mentioned in the discussion.

The authors are restating study results rather than interpreting them within a broader conceptual or theoretical context. The authors do not critically examine why or how monitoring mechanisms influenced dropout rates, nor do they acknowledge the methodological limitations of their study. As written, the discussion reads as confirmatory and, at times, overstates the study’s conclusions about effectiveness, which can be measured in an RCT. The authors should strengthen this section by providing a more analytical interpretation of findings, situating them within existing literature on immunization system performance, and explicitly addressing the study’s limitations and implications for policy and practice.

Although the authors include brief recommendations directed at policymakers within the conclusion, these points are not clearly separated or developed into a distinct section. The authors should consider presenting a concise “Implications for Policymakers” section that translates their findings into actionable guidance.

**********

what does this mean?). If published, this will include your full peer review and any attached files.

If you choose “no”, your identity will remain anonymous but your review may still be made public.

**Do you want your identity to be public for this peer review?**  For information about this choice, including consent withdrawal, please see our Privacy Policy

Reviewer #1: No

**********

[NOTE: If reviewer comments were submitted as an attachment file, they will be attached to this email and accessible via the submission site. Please log into your account, locate the manuscript record, and check for the action link "View Attachments". If this link does not appear, there are no attachment files.]

To ensure your figures meet our technical requirements, please review our figure guidelines: https://journals.plos.org/plosone/s/figures

You may also use PLOS’s free figure tool, NAAS, to help you prepare publication quality figures: https://journals.plos.org/plosone/s/figures#loc-tools-for-figure-preparation.

NAAS will assess whether your figures meet our technical requirements by comparing each figure against our figure specifications.

Attachment Submitted filename: Academic Editor Comments.docx

# Author comment: Author response to Decision Letter 1

**DOI:** 10.1371/journal.pone.0356268.r002

4 Jun 2026

i have created the ORCID id: 0009-0006-0809-1500

i have also adjusted the manuscript as per the guidelines shared in previous comments for revision

Response to Academic Editor Comments

Manuscript title: Effectiveness of Monitoring Mechanisms in Reducing Immunization Dropouts Among Infants in Health Facilities of Maridi County, South Sudan

Journal: PLOS ONE

Manuscript number: PONE-D-25-26100

Authors: Daniel Opinile Mark Igga, Nzomo Mwita, Kennedy Muni

Dear Editor,

We sincerely thank the Academic Editor for the careful and constructive review of our manuscript. The comments identified genuine weaknesses in clarity, precision, and organization that we believe were important to address. We have revised the manuscript thoroughly in response to every point raised. Below we provide a point-by-point response to each comment. For ease of reference, each editor comment is quoted verbatim (shaded grey), followed by our response and a description of the specific change made in the revised manuscript. Passages added or substantially rewritten in the manuscript are indicated in the third column.

We hope the revisions satisfy the editor’s concerns and that the manuscript is now suitable for publication in PLOS ONE.

Yours sincerely,

Daniel Opinile Mark Igga (on behalf of all authors)

Section 1: Abstract

Comment 1 — Abstract – Introduction

Editor Comment Authors’ Response Manuscript Change

"coverage reduced to 84%". At what time point? The editor is correct that the time point was missing, which made the statement imprecise and unverifiable. Global third-dose pentavalent coverage was 86% in 2019, declined to 84% in 2020, and fell further to 82% in 2021 — a three-year trend reported by the Immunization Agenda 2030. Specifying these years is essential for readers to contextualise the data against global benchmarks. Revised abstract (Introduction): "Third-dose pentavalent vaccine coverage stood at 86% globally in 2019, declining to 84% in 2020 and further to 82% in 2021, remaining below the 85% minimum target set by the Immunization Agenda 2030." All three years are now explicit.

Comment 2 — Abstract – Introduction

Editor Comment Authors’ Response Manuscript Change

"Despite the efforts employed to improve the immunization coverage, SUBJECT NEEDED HERE remained below 85%." — A subject is missing from this sentence. We agree. The sentence was grammatically incomplete. The intended subject was "immunization coverage," which had been dropped in editing. The sentence has been rewritten to remove the dangling clause entirely and incorporate the substance into a fuller, subject-complete sentence. Revised abstract (Introduction): The sentence now reads: "...declining to 84% in 2020 and further to 82% in 2021, consistently falling short of the 85% floor target set by the Immunization Agenda 2030." The incomplete clause has been eliminated.

Comment 3 — Abstract – Methods/Results

Editor Comment Authors’ Response Manuscript Change

"BHWs" — make sure the abbreviations are written out first time they are used. We agree. The abbreviation "BHW" appeared in the abstract without prior definition, which is inconsistent with PLOS ONE style and would confuse readers unfamiliar with South Sudan's community health architecture. The term is now spelled out in full on first use in the abstract, with the abbreviation provided in parentheses immediately afterward. Revised abstract (Results): "...Boma Health Workers (BHWs) — community-level health personnel who mobilize, trace, and refer immunization defaulters — were attached to 64% of facilities." Subsequent uses of "BHW/BHWs" throughout the abstract and manuscript are abbreviated.

Comment 4 — Abstract – Methods/Results

Editor Comment Authors’ Response Manuscript Change

Explain in abstract (briefly) what a Boma Health Worker is, also state how long the study lasted (i.e. over what period of time did changes occur?). Both points are well taken. First, the Boma Health Worker is a programme-specific cadre unique to South Sudan's health system, and readers outside the country would have no frame of reference for the term without a brief explanation. Second, without a time frame readers cannot judge how meaningful or durable the observed dropout reductions are. We have addressed both. Revised abstract (Methods): The study duration is now explicit: "...conducted from 1 October to 31 December 2023."

Revised abstract (Results): BHWs are defined inline: "Boma Health Workers (BHWs) — community-level health personnel who mobilize, trace, and refer immunization defaulters..."

Comment 5 — Abstract – Conclusions

Editor Comment Authors’ Response Manuscript Change

Can you distinguish between the monitoring tools used by internal health workers and the external monitoring and the effect of BHWs? This is an important conceptual distinction that the original abstract conflated. The monitoring chart, immunization register, and defaulter tracing system are facility-based (internal) tools used by vaccinators and health workers to track coverage and identify individual defaulters. BHWs operate at the community (external) level, mobilizing households, tracing missing children, and referring them back to facilities. The abstract conclusions have been rewritten to make this distinction explicit, so readers understand that the two mechanisms are complementary rather than interchangeable. Revised abstract (Conclusions): "Both facility-based tools (monitoring charts, defaulter tracing systems) and community-based mechanisms (BHWs) are independently associated with lower immunization dropout rates, and their combination appears to have a complementary effect." The distinction between internal and external monitoring is now explicit in the conclusions.

Section 2: Introduction

Comment 6 — Introduction

Editor Comment Authors’ Response Manuscript Change

"The number of infants vaccinated globally reduced, specifically, uptake of 3rd dose of pentavalent." Statements like this need to have a time frame. The next sentence is not clear. We agree on both counts. The original statement was both imprecise (no year specified) and syntactically awkward ("specifically, uptake of 3rd dose of pentavalent" does not complete a grammatical thought). The subsequent sentence about the Immunization Agenda 2030 target was also unclear in its relationship to the preceding figures. We have completely rewritten this passage with explicit years (2019, 2020, 2021) drawn from the Immunization Agenda 2030 global data, and the relationship between the observed decline and the target threshold is now stated directly. Revised Introduction: "Third-dose pentavalent coverage, a widely used proxy for immunization system performance, stood at 86% globally in 2019 but slipped to 84% in 2020 and 82% in 2021, consistently falling short of the 85% floor target set by the Immunization Agenda 2030." The unclear follow-on sentence has been removed and replaced with a direct statement about what falling short of the target means for program planning.

Comment 7 — Introduction

Editor Comment Authors’ Response Manuscript Change

"The study intends to determine the use of immunization monitoring mechanism as a strategy to improve the county's performance of 3rd dose of pentavalent, according to 2022 data, which is 82% and the quite low measles vaccine coverage at 55% to the minimum target of 85% through the proper use of immunization monitoring mechanisms." This sentence is repetitive about using monitoring mechanisms. We agree that the phrase "immunization monitoring mechanism" appeared twice in the same sentence and the sentence was also structurally unwieldy. The entire passage has been reorganised. The Maridi County coverage figures (82% pentavalent-3 and 55% measles in 2022) now appear in a standalone sentence providing factual context, and the study aim is stated separately and more concisely. Revised Introduction: The county's coverage gap is stated as: "...2022 programme data revealed 82% third-dose pentavalent coverage and 55% measles vaccine coverage in the county — both below the national 85% target." The study purpose is then stated separately without repetition of the phrase "monitoring mechanisms."

Comment 8 — Introduction

Editor Comment Authors’ Response Manuscript Change

Why "Nevertheless"? It seems like it's all saying the same thing. The editor is right. "Nevertheless" implies a contrast or concession, but the surrounding sentences were building the same argument rather than pivoting to a contrasting point. The word was being used as a filler transition. The paragraph has been restructured so that each sentence advances a distinct idea: (1) the coverage gap in Maridi County, (2) what the target is, (3) why monitoring and community tracing matter, and (4) the study aim. The transitional word "Nevertheless" has been removed entirely. Revised Introduction: The paragraph has been restructured. "Nevertheless" has been deleted, and the sentences that followed it have been reorganised to flow logically without a false contrast marker.

Comment 9 — Introduction

Editor Comment Authors’ Response Manuscript Change

"monthly goals on a timely fashion, provide basis for…" should be "in a timely fashion" and "provides" and "reflecting". We thank the editor for catching these grammatical errors. "On a timely fashion" is incorrect idiom; the correct phrase is "in a timely fashion." The verb "provide" should agree with its singular subject ("this tool") and read "provides." "Reflect" in the participial phrase should read "reflecting" for grammatical parallelism. All three corrections have been made. Revised Introduction: The sentence describing the monitoring chart now reads: "...set and evaluate monthly goals in a timely fashion, provides a basis for comparing performance across time periods...and reflects the situation accurately." All three grammatical corrections are incorporated.

Section 3: Materials and Methods

Comment 10 — Methods

Editor Comment Authors’ Response Manuscript Change

"vaccinator was selected based on toasted coin" should be based on a tossed coin or based on a coin toss. This is a clear spell-checker substitution error ("toasted" for "tossed") that we missed in proofreading. We apologize for the oversight. The phrase has been corrected throughout the manuscript. Revised Methods (Sampling): "...one was enrolled in the study by coin toss, yielding 13 participants per stratum (26 total)." All instances of "toasted coin" have been replaced with "coin toss."

Comment 11 — Methods

Editor Comment Authors’ Response Manuscript Change

"had a written informed consent, that" — no comma should be here between subject and verb. Correct. The comma between "consent" and "that" incorrectly separates the noun phrase from its relative clause. The comma has been removed. The sentence has also been further revised for clarity (see Comment 12 below). Revised Methods (Data Collection): The comma has been removed. The sentence now reads: "...a semi-structured interview guide that included a written informed consent section..."

Comment 12 — Methods

Editor Comment Authors’ Response Manuscript Change

"This examined whether the presence of BHWs in health facilities was either attributed to having low dropout rates (less than 10%) or high dropout rates (more than 10%)." Perhaps rather than "attributed to," you meant to say "associated with." The sentence now seems to say that the dropout rate caused the BHW to be in the health facility. This is an important clarification. The original phrasing was causally ambiguous — it could imply that authorities deployed BHWs to facilities because those facilities already had high dropout rates (i.e., dropout rate caused BHW presence), when in fact we were testing the reverse hypothesis (BHW presence influences dropout rate). The correct statistical framing is one of association, not attribution. We have replaced "attributed to" with "associated with" throughout the manuscript and added a brief note in the Discussion clarifying that the study design does not permit causal inference. Revised Methods (Analysis): "Chi-square tests were used to examine whether BHW presence in health facilities was associated with lower dropout rates (below 10%) or higher dropout rates (above 10%)." The word "attributed" has been replaced with "associated" throughout.

Comment 13 — Methods

Editor Comment Authors’ Response Manuscript Change

"The researcher provided written informed consent to the participants." It seems that the researcher OBTAINED written informed consent FROM the participants. What was the original language of the informed consent? Please state whether the IC translation was written (and verified in some way and approved by ethics committee) or by a script that was oral (approved?), or whether it was done each time verbally by the person obtaining informed consent. The editor is entirely correct, and we apologize for the imprecise language. The researcher obtained written informed consent from the participants — not the other way around. Additionally, we recognize that the original manuscript failed to specify the informed consent language protocol, which is an important omission for a study conducted in a multilingual, low-resource setting.

To clarify: The informed consent form was prepared in English. Because many participants are more comfortable in local languages, the researcher verbally interpreted the consent form into the participant's preferred language — Juba Arabic, Muru, Azande, Baka, or Avokaya — prior to the participant signing. This verbal translation protocol was reviewed and approved by the AMIU Ethics and Scientific Review Committee and the South Sudan MOH Research Ethics Review Board as part of the study ethics application. Consent was obtained in writing from every participant; the signature confirmed that the verbal explanation had been understood and that the participant agreed voluntarily to take part. Revised Methods (Ethics): "Written informed consent was obtained from every enrolled vaccinator before data collection began. The consent form was prepared in English and verbally interpreted by the researcher into each participant's preferred language (Juba Arabic, Muru, Azande, Baka, or Avokaya) prior to signature. This verbal interpretation protocol was reviewed and approved as part of the study's ethics application to the AMIU ESRC and the South Sudan MOH-RERB."

Section 4: Results

Comment 14 — Results – Formatting

Editor Comment Authors’ Response Manuscript Change

"Out of the 14 health facilities, (12, 86%) were supported…" The 12 should be before parentheses. Same for the 2 later on in the sentence, etc. We agree. Placing the count outside the parentheses and the percentage inside is the correct and widely adopted convention in biomedical writing — e.g., "12 (86%)" rather than "(12, 86%)". This formatting error appeared throughout the original Results section. All instances have been corrected. Revised Results: All frequency-percentage pairs are now formatted as "n (%)" throughout — e.g., "12 (86%)," "3 (21%)," "13 (93%)" — consistent with PLOS ONE style.

Comment 15 — Results – Demographics

Editor Comment Authors’ Response Manuscript Change

"Majority of the male respondents (11, 79%)…" Be clear that you mean by respondent the person responding on behalf of the health facility. We agree that the term "respondent" was ambiguous and could be read as referring to a member of the general public. Since each health facility contributed one vaccinator to the study, the "respondent" is always the vaccinator selected to represent that facility. We have clarified this at first use and maintained the clarification consistently throughout the Results section. Revised Results (Demographics): "...14 respondents (one vaccinator per facility, selected to represent their health facility)." Subsequent mentions describe respondents as "vaccinators" or "health facility representatives" to avoid ambiguity.

Comment 16 — Results – Demographics

Editor Comment Authors’ Response Manuscript Change

The % of males who had secondary education seems to be 100%. The % of all respondents who had secondary education is 79%. Does this mean there were just 3 women responding? Make that respondentS. The editor is corre

Attachment Submitted filename: Response_to_Editor_Comments.docx

# Editor report: Decision Letter 1

**DOI:** 10.1371/journal.pone.0356268.r003

**Authors:** Patricia Fast

15 Jun 2026

Dear Dr. Igga,

Thank you for submitting your manuscript to PLOS ONE. After careful consideration, we feel that it has merit but does not fully meet PLOS ONE’s publication criteria as it currently stands. Therefore, we invite you to submit a revised version of the manuscript that addresses the points raised during the review process.

Please submit your revised manuscript by Jul 30 2026 11:59PM. If you will need more time than this to complete your revisions, please reply to this message or contact the journal office at plosone@plos.org. When you're ready to submit your revision, log on to https://www.editorialmanager.com/pone/ and select the 'Submissions Needing Revision' folder to locate your manuscript file.

- A letter that responds to each point raised by the academic editor and reviewer(s). You should upload this letter as a separate file labeled 'Response to Reviewers'.
- A marked-up copy of your manuscript that highlights changes made to the original version. You should upload this as a separate file labeled 'Revised Manuscript with Track Changes'.
- An unmarked version of your revised paper without tracked changes. You should upload this as a separate file labeled 'Manuscript'.

If you would like to make changes to your financial disclosure, please include your updated statement in your cover letter. Guidelines for resubmitting your figure files are available below the reviewer comments at the end of this letter.

If applicable, we recommend that you deposit your laboratory protocols in protocols.io to enhance the reproducibility of your results. Protocols.io assigns your protocol its own identifier (DOI) so that it can be cited independently in the future. For instructions see: https://journals.plos.org/plosone/s/submission-guidelines#loc-laboratory-protocols. Additionally, PLOS ONE offers an option for publishing peer-reviewed Lab Protocol articles, which describe protocols hosted on protocols.io. Read more information on sharing protocols at https://plos.org/protocols?utm_medium=editorial-email&utm_source=authorletters&utm_campaign=protocols.

As the corresponding author, your ORCID iD is verified in the submission system and will appear in the published article. PLOS supports the use of ORCID, and we encourage all coauthors to register for an ORCID iD and use it as well. Please encourage your coauthors to verify their ORCID iD within the submission system before final acceptance, as unverified ORCID iDs will not appear in the published article. *Only*  the individual author can complete the verification step; PLOS staff *cannot*  verify ORCID iDs on behalf of authors.

We look forward to receiving your revised manuscript.

Kind regards,

Patricia Evelyn Fast, MD, Ph.D.

Academic Editor

PLOS One

Journal Requirements:

If the reviewer comments include a recommendation to cite specific previously published works, please review and evaluate these publications to determine whether they are relevant and should be cited. There is no requirement to cite these works unless the editor has indicated otherwise.

[Note: HTML markup is below. Please do not edit.]

[NOTE: If reviewer comments were submitted as an attachment file, they will be attached to this email and accessible via the submission site. Please log into your account, locate the manuscript record, and check for the action link "View Attachments". If this link does not appear, there are no attachment files.]

To ensure your figures meet our technical requirements, please review our figure guidelines: https://journals.plos.org/plosone/s/figures

You may also use PLOS’s free figure tool, NAAS, to help you prepare publication quality figures: https://journals.plos.org/plosone/s/figures#loc-tools-for-figure-preparation.

NAAS will assess whether your figures meet our technical requirements by comparing each figure against our figure specifications.

Attachment Submitted filename: Reviewer.docx

# Author comment: Author response to Decision Letter 2

**DOI:** 10.1371/journal.pone.0356268.r004

29 Jul 2026

Comments addressed in the uploaded file Response to Editor Comments

Attachment Submitted filename: Response to Editor Comments.docx

# Editor report: Decision Letter 2

**DOI:** 10.1371/journal.pone.0356268.r005

**Authors:** Patricia Fast

3 Aug 2026

EFFECTIVENESS OF MONITORING MECHANISMS IN REDUCING IMMUNIZATION DROPOUTS AMONG INFANTS IN HEALTH FACILITIES OF MARIDI COUNTY, SOUTH SUDAN

PONE-D-25-26100R2

Dear Dr. Igga:

We’re pleased to inform you that your manuscript has been judged scientifically suitable for publication and will be formally accepted for publication once it meets all outstanding technical requirements.

Within one week, you’ll receive an e-mail detailing the required amendments. When these have been addressed, you’ll receive a formal acceptance letter and your manuscript will be scheduled for publication.

An invoice will be generated when your article is formally accepted. Please note, if your institution has a publishing partnership with PLOS and your article meets the relevant criteria, all or part of your publication costs will be covered. Please make sure your user information is up-to-date by logging into Editorial Manager at Editorial Manager® and clicking the ‘Update My Information' link at the top of the page. For questions related to billing, please contact billing support.

If your institution or institutions have a press office, please notify them about your upcoming paper to help maximize its impact. If they’ll be preparing press materials, please inform our press team as soon as possible -- no later than 48 hours after receiving the formal acceptance. Your manuscript will remain under strict press embargo until 2 pm Eastern Time on the date of publication. For more information, please contact onepress@plos.org.

Kind regards,

Patricia Evelyn Fast, MD, Ph.D.

Academic Editor

PLOS One

Additional Editor Comments (optional):

Thank you for your diligence in addressing the reviews. Please note that one issue seems to remain: The following statement seems inconsistent with the data in the table re: educational level. " Ten respondents (71%) had reached

secondary level of education and 4 (29%) primary level. Disaggregated by sex, all 11 male

respondents had attained secondary-level education, while all 3 female respondents had attained

primary-level education only. "

Reviewers' comments:

Ten respondents (71%) had reached

secondary level of education and 4 (29%) primary level. Disaggregated by sex, all 11 male

respondents had attained secondary-level education, while all 3 female respondents had attained

primary-level education only.

# Editor report: Acceptance letter

**DOI:** 10.1371/journal.pone.0356268.r006

**Authors:** Patricia Fast

PONE-D-25-26100R2

PLOS One

Dear Dr. Igga,

I'm pleased to inform you that your manuscript has been deemed suitable for publication in PLOS One. Congratulations! Your manuscript is now being handed over to our production team.

At this stage, our production department will prepare your paper for publication. This includes ensuring the following:

* All references, tables, and figures are properly cited

* All relevant supporting information is included in the manuscript submission,

* There are no issues that prevent the paper from being properly typeset

You will receive further instructions from the production team, including instructions on how to review your proof when it is ready. Please keep in mind that we are working through a large volume of accepted articles, so please give us a few days to review your paper and let you know the next and final steps.

Lastly, if your institution or institutions have a press office, please let them know about your upcoming paper now to help maximize its impact. If they'll be preparing press materials, please inform our press team within the next 48 hours. Your manuscript will remain under strict press embargo until 2 pm Eastern Time on the date of publication. For more information, please contact onepress@plos.org.

You will receive an invoice from PLOS for your publication fee after your manuscript has reached the completed accept phase. If you receive an email requesting payment before acceptance or for any other service, this may be a phishing scheme. Learn how to identify phishing emails and protect your accounts at https://explore.plos.org/phishing.

If we can help with anything else, please email us at customercare@plos.org.

Thank you for submitting your work to PLOS One and supporting open access.

Kind regards,

PLOS One Editorial Office Staff

on behalf of

Dr. Patricia Evelyn Fast

Academic Editor

PLOS One
